# Supplementary material for: SAMHD1 Limits the Efficacy of Forodesine in Leukemia by Protecting Cells against the Cytotoxicity of dGTP
Source: Cell Rep. 2020 May 12;31(6):107640. doi: 10.1016/j.celrep.2020.107640 (PMC7225753; doi:10.1016/j.celrep.2020.107640)
Supplement: Document S1. Figures S1–S6 and Table S1 [file mmc1.pdf]

Cell Reports, Volume *31*

Supplemental Information

SAMHD1 Limits the Efficacy of Forodesine in Leukemia by Protecting Cells against the Cytotoxicity of dGTP

Tamara Davenne, Jenny Klintman, Sushma Sharma, Rachel E. Rigby, Henry T.W. Blest, Chiara Cursi, Anne Bridgeman, Bernadeta Dadonaite, Kim De Keersmaecker, Peter Hillmen, Andrei Chabes, Anna Schuh, and Jan Rehwinkel

Figure S1

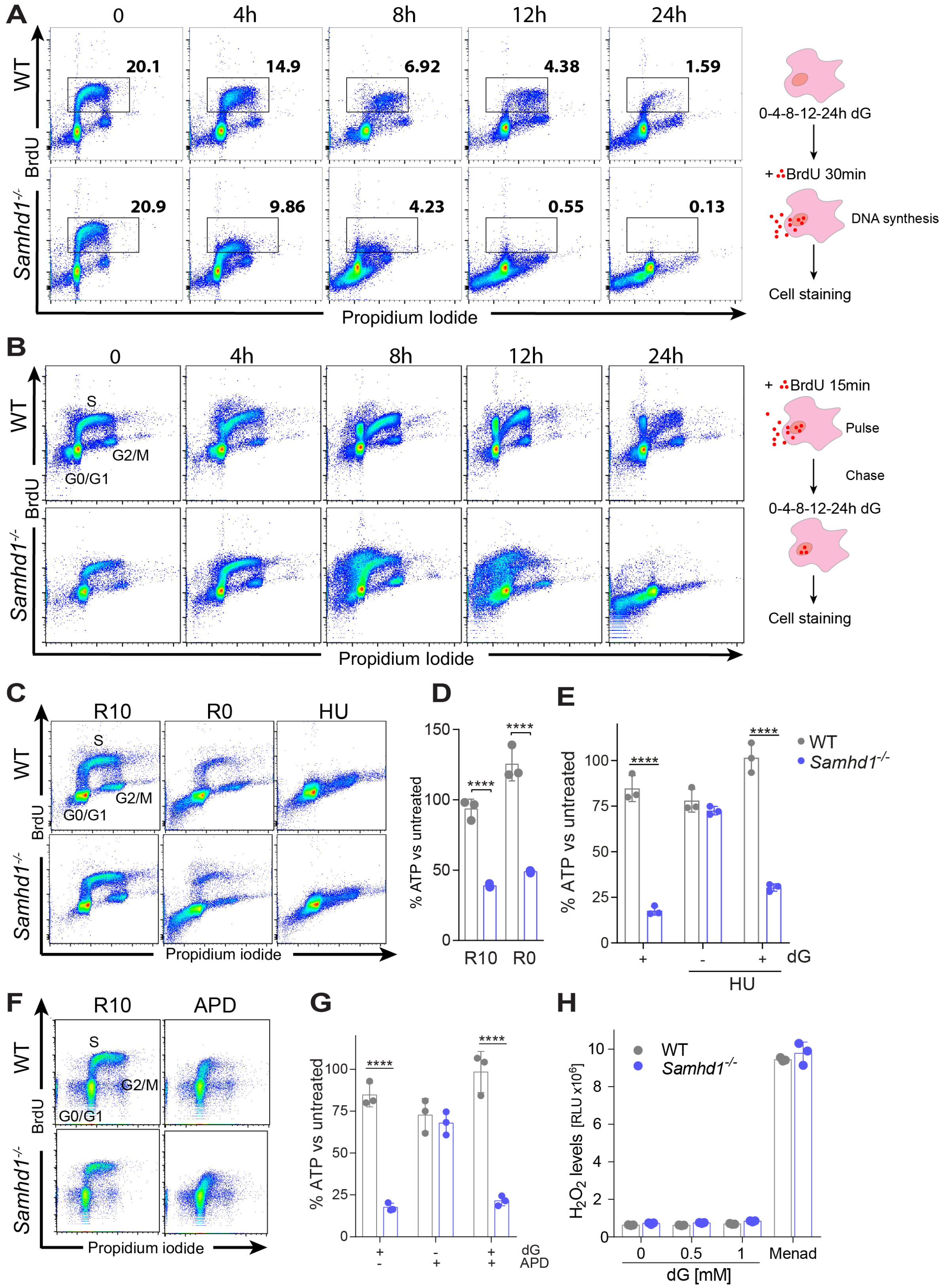

Figure S1. dG-induced cell death in *Samhd1*$^{-/-}$ cells is independent of nuclear DNA replication, related to Figure 2.

(**A**) BMDMs were treated with 0.5 mM dG for the indicated periods of time. Cells were then labelled with BrdU for 30 minutes and fixed. Cells were stained using α-BrdU antibody and PI and analysed by flow cytometry.
(**B**) BMDMs were labelled with BrdU for 15 minutes and then treated with 0.5 mM dG for the indicated periods of time. Cells were then analysed as in (**A**).
(**C-E**) After 7 days of conventional culture, BMDMs were grown in medium containing 10% FCS (R10) or in serum-free medium (R0) for 24 hours. Alternatively, BMDMs were treated with 1 mM hydroxyurea (HU) for 8 hours. (**C**) Cells were analysed as in (**A**). (**D**) Cells were treated with 0.5 mM dG for 24 hours and viability was analysed as described in Figure 1A. (**E**) Cells were treated with 0.5 mM dG for 16 hours and viability was analysed as described in Figure 1A.
(**F-G**) BMDMs were treated or not with 1 μM of aphidicolin (APD) in R10 for 16 hours. (**F**) Cells were analysed as in (**A**). (**G**) Cells were treated with 0.5 mM dG for 24 hours and viability was analysed as described in Figure 1A.
(**H**) H_2O_2 production was measured using the ROS-Glo H_2O_2 assay (Promega). BMDMs were treated with the indicated concentrations of dG for 3 hours. The H_2O_2 substrate solution was then added for 6 hours. Control cells were treated with 20 μM Menadione (Menad) during this period. Luminescence was measured after addition of the detection solution.
Data are representative of three (**A-G**) or two (**H**) independent experiments, respectively. In panels **D-E** and **G-H** data from triplicate measurements are shown with mean ± SD. P-values determined with two-way ANOVA are indicated. **** $p<0.0001$.

Figure S2

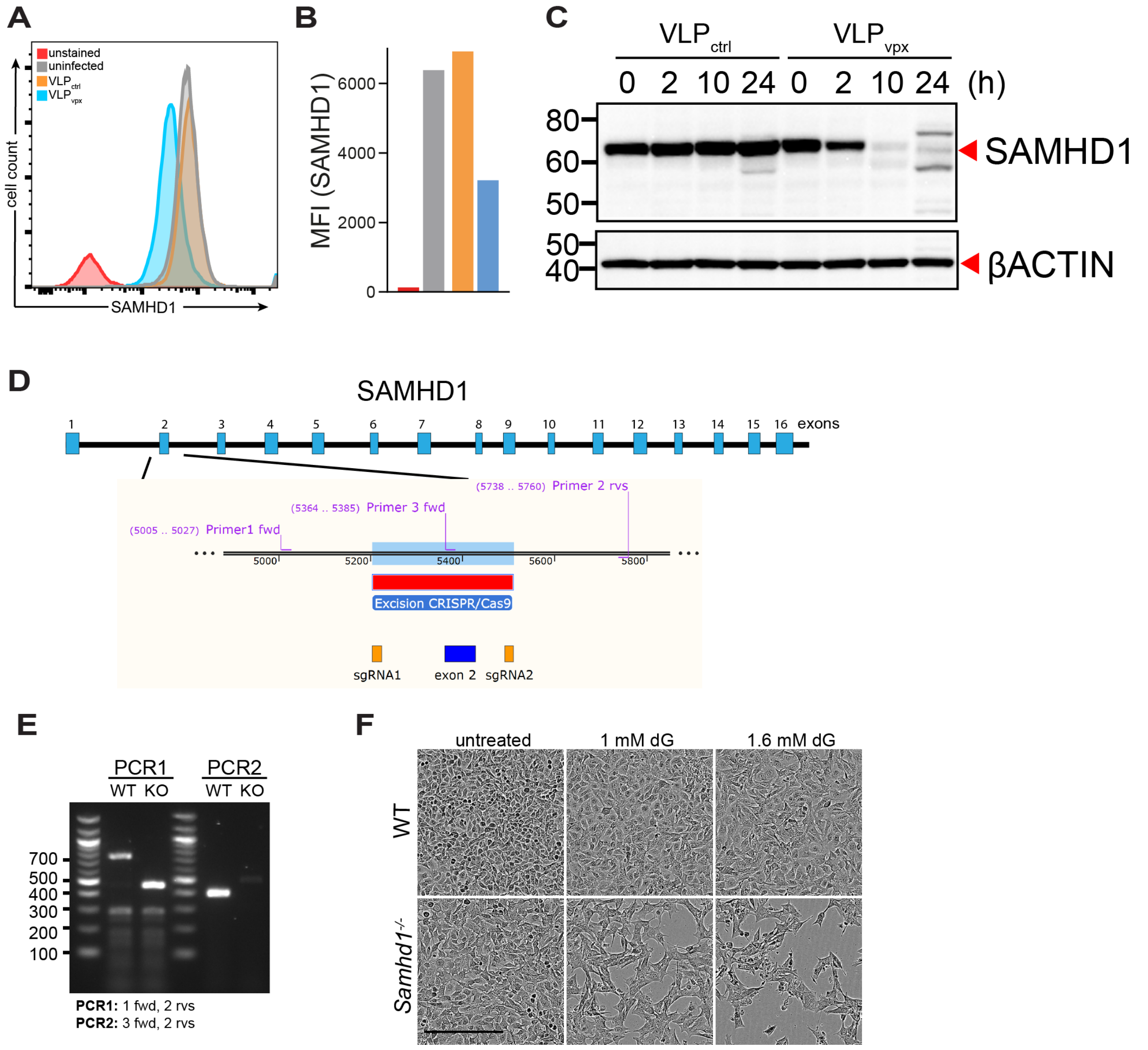

Figure S2. Validation of SAMHD1-deficient cells, related to Figure 3.

(**A,B**) HeLa treated as described in Figure 3A,B were stained with α-SAMHD1 antibody and analysed by flow cytometry. Data are represented as histograms (**A**) and the mean fluorescence intensity (MFI) of the SAMHD1 signal is shown in (**B**).

(C) MDA-MB231 cells were infected with VLPs containing Vpx (VLP_{vpx}) or not (VLP_{ctrl}). SAMHD1 levels in total cell extracts were determined by western blot at the indicated time-points after infection. β-Actin served as a loading control.

(**D,E**) Representation of the CRISPR/Cas9 strategy to generate B16F10 *Samhd1*$^{-/-}$ cells used in Figure 3E-G. The knock-out of *Samhd1* exon 2 was validated by PCR using the indicated primers (**E**).

(**F**) Brightfield images of WT and *Samhd1*$^{-/-}$ B16F10 cells 20 hours after treatment with dG from the experiment in Figure 3G are shown. The scale bar represents 300 μm. Panels **A-C** and **F** are representative of three independent experiments, respectively.

Figure S3

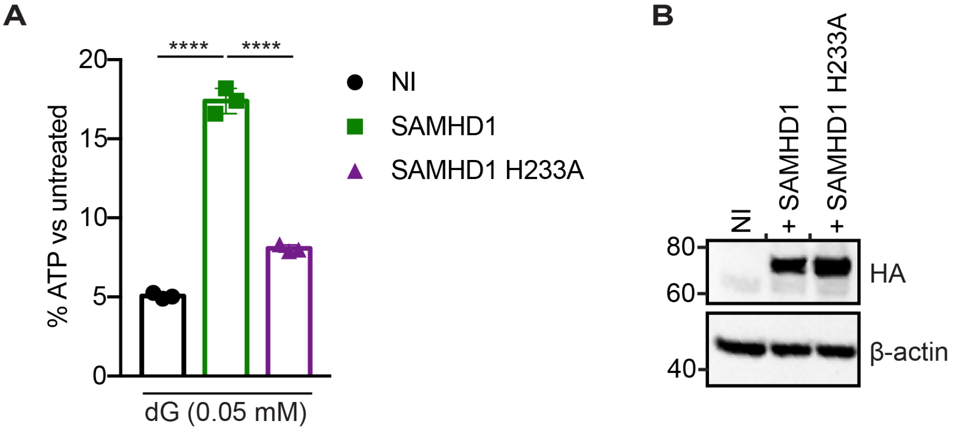

Figure S3. SAMHD1's dNTPase activity is required to protect cells against dG-induced toxicity, related to Figure 3.

(**A**,**B**) Jurkat cells were reconstituted with HA-tagged wild-type or H233A mutant SAMHD1 using a lentivector. Un-infected cells (NI) served as control. (**A**) Cells were then treated with 0.05 mM dG for 48 hours. Cell viability was determined as in Figure 1A. (**B**) SAMHD1 levels in total cell extracts were determined by western blot. β-Actin served as a loading control.

Data are representative of two independent experiments. In panel A, data from triplicate measurements are shown with mean ± SD. P-values determined with two-way ANOVA are indicated. **** $p<0.0001$.

Figure S4

A

Sample ID	Age	Gender	*Del17p*	*TP53*	*SAMHD1*	*del11q (ATM)*	*IgVH status*	Group
ADM224	41	M				y	unmutated	*SAMHD1*^{nonmut}
ADM295	68	M				y	unmutated	
ARC138	66	M				y	unmutated	
ARC554	70	M				y	mutated	
ADM013	72	M		y			missing	*SAMHD1*^{nonmut}
ADM020	69	M		y			unmutated	
ARC334	75	M		y			unmutated	
ADM181	68	M	y	y			unmutated	
ADM216	62	M	y	y			mutated	
ARC603	62	M	y	y			unmutated	
ADM007	59	M			y		unmutated	*SAMHD1*^{mut}
ADM069	59	M			y		unmutated	
ADM210	55	M			y		mutated	
ADM228	57	M			y		unmutated	

B

Sample ID	Exon	Nucleotide	Amino Acid	VAF	SAMHD1 locus
▲ ADM007	exon5	T602A	I201N	0.081	cnLOH
ADM007	exon5	A617G	H206R	0.176	cnLOH
ADM007	exon11	G1165A	A389T	0.492	cnLOH
● ADM069	exon4	G434A	R145Q	0.958	cnLOH
■ ADM210	exon15	1694_1705del	565_569del	0.935	CNLoss
◆ ADM228	exon15	G1715A	W572X	0.982	cnLOH

C

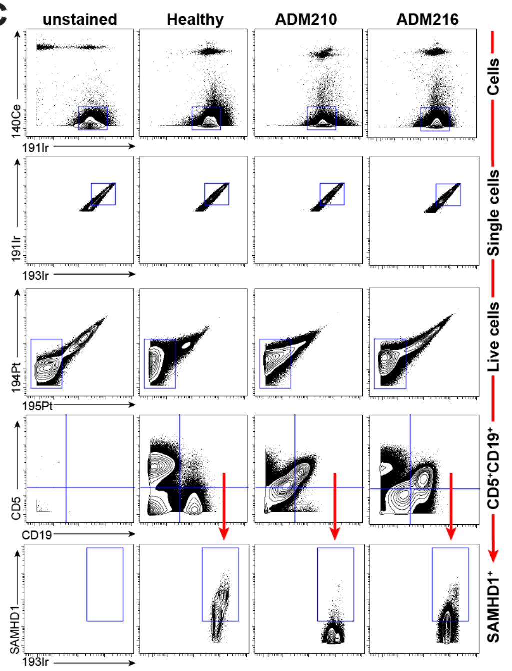

D

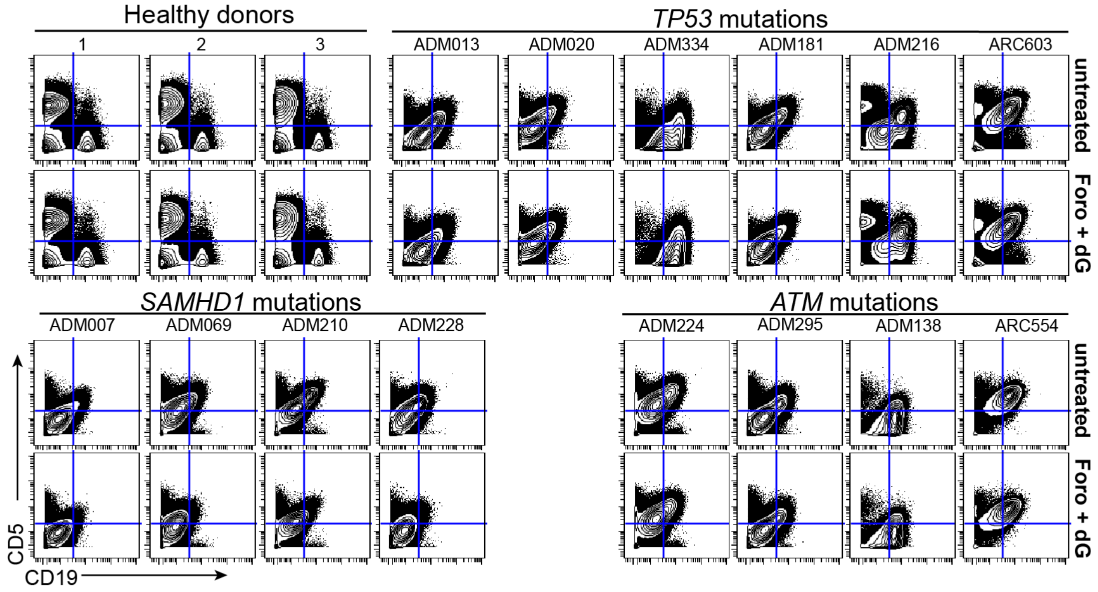

Figure S4. CyTOF analysis, related to Figure 5.

(**A**) List of CLL samples, age of patient at sample collection and mutation status. CLL samples carrying either *TP53* lesions (Del17p or mutation) or *ATM* lesions (Del11q) served as controls for *SAMHD1* mutated samples. M, male; IgVH, immunoglobulin variable heavy chain gene.

(**B**) Details of *SAMHD1* mutations. VAF, Variant allele frequency; cnLOH, copy neutral loss of heterozygosity; CNLoss, copy number loss. The geometrical shapes shown in blue are used in Figure 5 (panels A, C, D, F-H) to identify samples.

(**C**) CyTOF gating strategy for the experiment shown in Figure 5. In brief, the 140Ce channel was used to exclude calibration beads. The 191Ir and 193Ir channels detect cells labelled with an intercalator and were used to identify single cells. The 194Pt and 195Pt channels were used to remove dead (cisplatin-labelled) cells from subsequent analysis of CD5, CD19 and SAMHD1 expression.

(**D**) Expression of CD5 and CD19 is shown as in Figure 5B for all analysed samples.

Figure S5

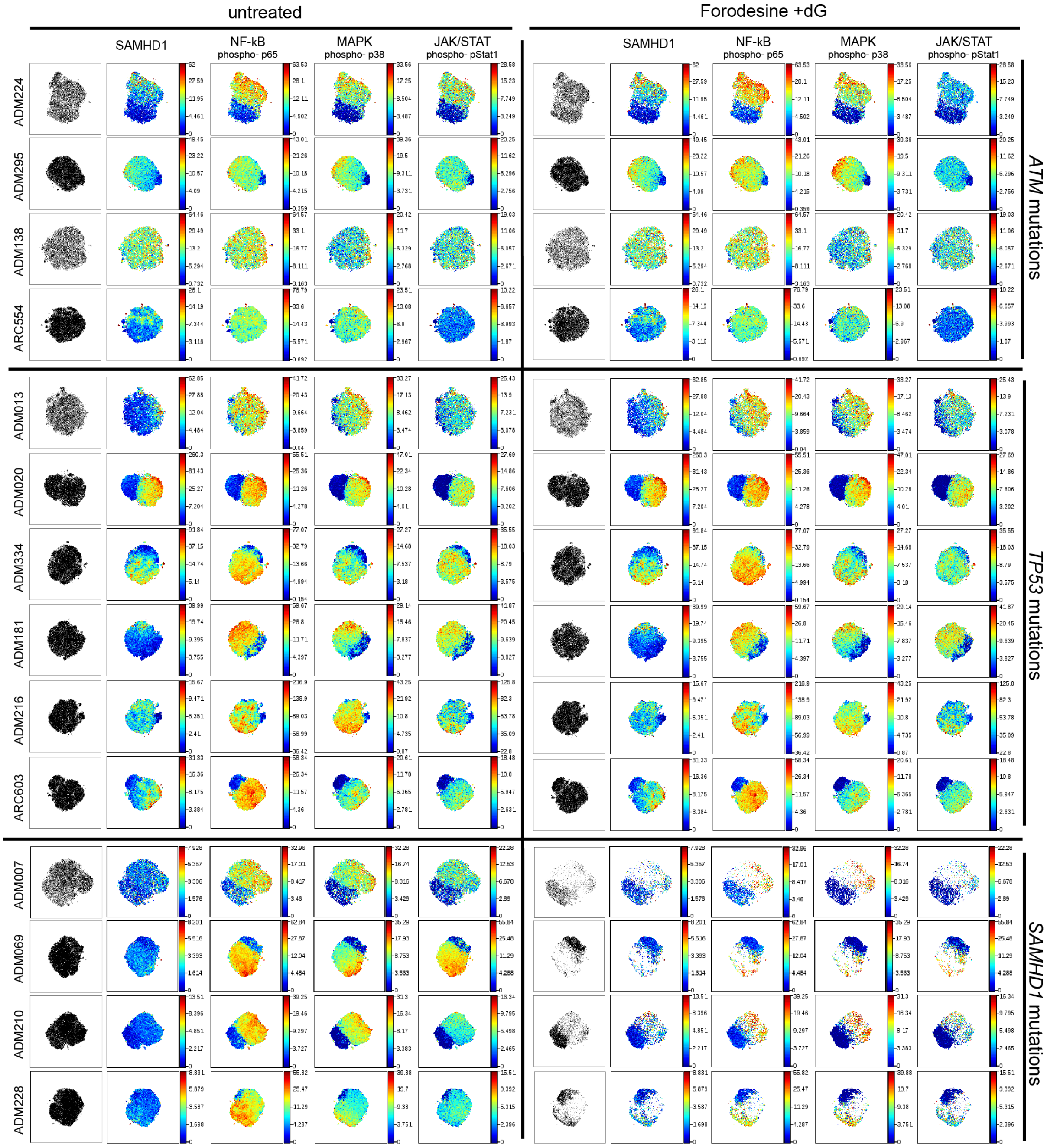

Figure S5. CLL patient cells with *SAMHD1* mutation are more sensitive to Forodesine and dG treatment, related to Figure 5.

CyTOF data from each sample were analysed separately by viSNE analysis as described in Figure 5E. Data from all patients studied are shown; for completeness, we included here those selected for Figure 5E.

Figure S6

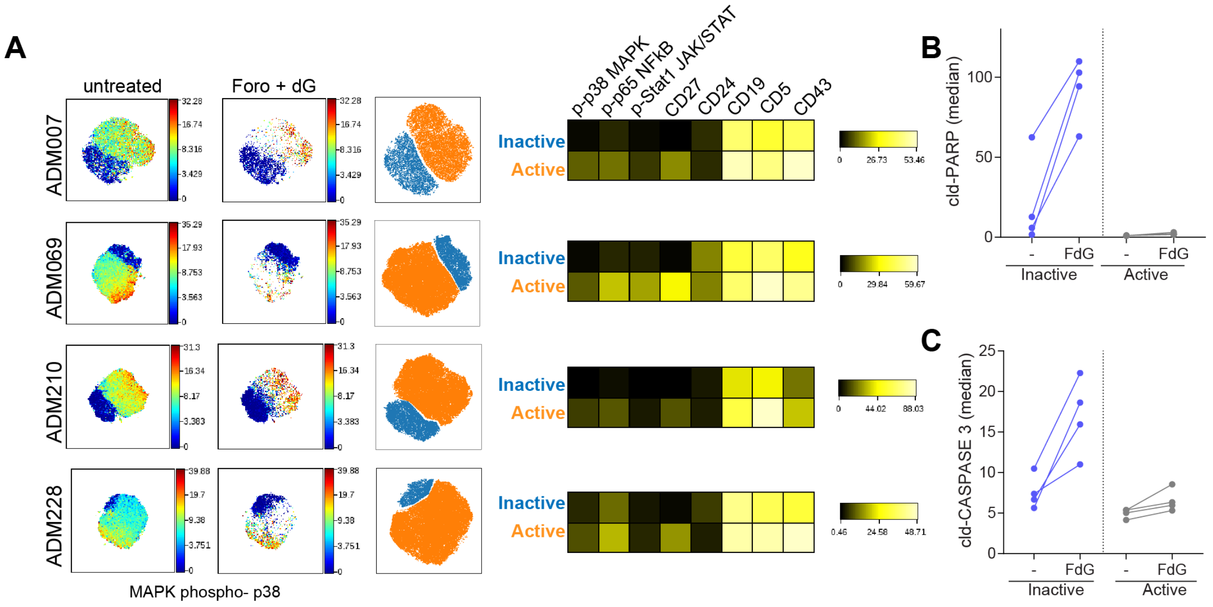

Figure S6. Forodesine and dG induce apoptosis in “inactive” CLL B-cells, related to Figure 5.

(**A**) tSNE plots using live CD5$^+$CD19$^+$ cells from *SAMHD1* mutated patients, coloured by p-p38 levels, were used to manually gate “inactive” [blue] and “active” [orange] cells (left). Expression of selected markers in these sub-populations is shown as a heat map (right).

(**B,C**) The staining for cleaved-PARP (**B**) and cleaved-CASPASE3 (**B**) was analysed in “inactive” and “active” cells. Median values are shown in untreated and treated cells. FdG, treatment with 2 μM forodesine and 20 μM dG.

Table S1. CyTOF antibody panel, related to STAR methods.

Target	Clone	Company	Catalog	Location
CD45	HI30	Fluidigm	3089003B	surface
CD11c	Bu15	Biolegend	337221	surface
CD11b (Mac-1)	ICRF44	Biolegend	301337	surface
CD4	RPA-T4	Fluidigm	3145001B	surface
CD20	2H7	Fluidigm	3147001B	surface
CD56 (NCAM)	NCAM16.2	Fluidigm	3149021B	surface
CD123	6H6	Biolegend	306027	surface
CD27	O323	Biolegend	302839	surface
CD38	HIT2	Biolegend	303535	surface
CD45RA	HI-100	Biolegend	304143	surface
CD14	M5E2	Fluidigm	3160001B	surface
CD23	EBVCS-5	Fluidigm	3164018B	surface
CD197 (CCR7)	G043H7	Fluidigm	3167009A	surface
CD8a	SK1	Fluidigm	3168002B	surface
CD24	ML5	Fluidigm	3169004B	surface
CD3	UCHT1	Fluidigm	3170001B	surface
CD19	HIB19	Biolegend	302247	surface
CD5	UCHT2	Biolegend	300627	surface
HLA-DR	L243	Fluidigm	3174001B	surface
CD194 (CCR4)	205410	Fluidigm	3175021A	surface
CD43	eBio84-3C1	Invitrogen	14-0439-82	surface
CD16	3G8	Fluidigm	3209002B	surface
cld-CASP3	5AE1	Fluidigm	3172023A	intracellular
cld-PARP	F21-852	Fluidigm	3143011C	intracellular
pStat1 [Y701]	4a	Fluidigm	3153005A	intracellular
p38 [T180/Y182]	D3F9	Fluidigm	3156002A	intracellular
SAMHD1	polyclonal	Abcam	Ab67820	intracellular
pNFκBp65 [S529]	K10x	Fluidigm	3166006A	intracellular
