# Supplementary material for: SAMHD1 Limits the Efficacy of Forodesine in Leukemia by Protecting Cells against the Cytotoxicity of dGTP
Source: Cell Rep. 2020 May 12;31(6):107640. doi: 10.1016/j.celrep.2020.107640 (PMC7225753; doi:10.1016/j.celrep.2020.107640)
Supplement: Document S2. Article plus Supplemental Information [file mmc2.pdf]

Cell Reports

Article

SAMHD1 Limits the Efficacy of Forodesine in Leukemia by Protecting Cells against the Cytotoxicity of dGTP

Graphical Abstract

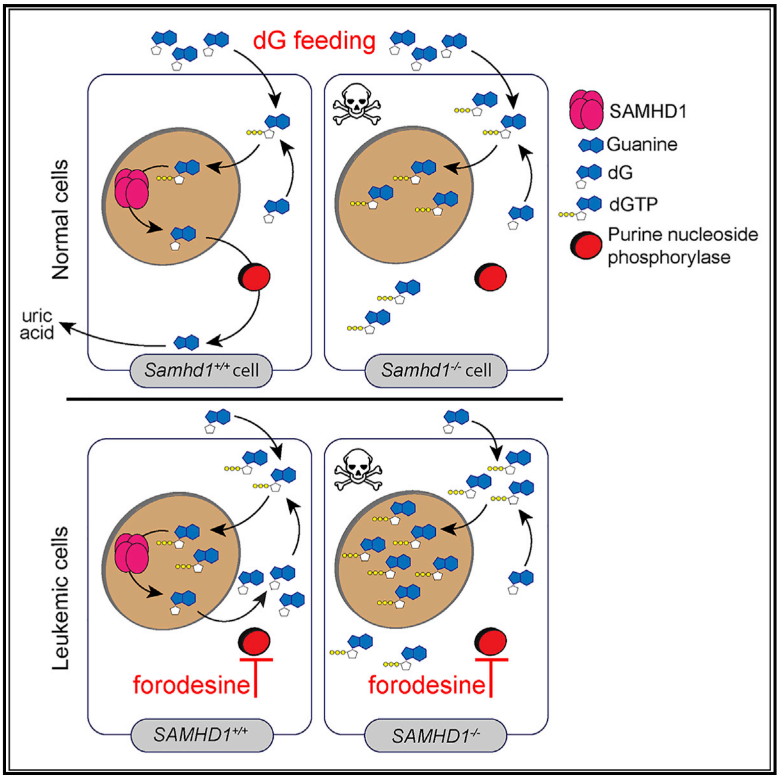

Highlights

- SAMHD1-deficient cells die upon exposure to deoxyguanosine (dG)
- dG induces apoptosis in cells, including cancer cells, lacking SAMHD1
- PNP-inhibitors such as forodesine and dG synergistically trigger cell death
- dG and forodesine kill mutated leukemic cells without SAMHD1 expression

Authors

Tamara Davenne, Jenny Klintman, Sushma Sharma, ..., Andrei Chabes, Anna Schuh, Jan Rehwinkel

Correspondence

jan.rehwinkel@imm.ox.ac.uk

In Brief

SAMHD1 degrades deoxyribonucleoside triphosphates (dNTPs), the building blocks of DNA. Davenne et al. find that SAMHD1 protects cells against dNTP imbalances. Exposure of SAMHD1-deficient cells to deoxyguanosine (dG) results in increased intracellular dGTP levels and subsequent apoptosis. This can be exploited to selectively kill cancer cells that acquired *SAMHD1* mutations.

Davenne et al., 2020, Cell Reports *31*, 107640
May 12, 2020 © 2020 The Author(s).
https://doi.org/10.1016/j.celrep.2020.107640

CellPress

Cell Reports

CellPress
OPEN ACCESS

Article

SAMHD1 Limits the Efficacy of Forodesine in Leukemia by Protecting Cells against the Cytotoxicity of dGTP

Tamara Davenne,[1,5] Jenny Klintman,[2] Sushma Sharma,[3] Rachel E. Rigby,[1] Henry T.W. Blest,[1] Chiara Cursi,[1] Anne Bridgeman,[1] Bernadeta Dadonaite,[4] Kim De Keersmaecker,[5] Peter Hillmen,[6] Andrei Chabes,[3] Anna Schuh,[2,7,8] and Jan Rehwinkel[1,9,*]

[1]Medical Research Council Human Immunology Unit, Medical Research Council Weatherall Institute of Molecular Medicine, Radcliffe Department of Medicine, University of Oxford, Oxford OX3 9DS, UK

[2]Molecular Diagnostic Centre, Department of Oncology, University of Oxford, Oxford OX3 7DQ, UK

[3]Department of Medical Biochemistry and Biophysics and Laboratory for Molecular Infection Medicine Sweden (MIMS), Umea University, 901 87 Umea, Sweden

[4]Sir William Dunn School of Pathology, University of Oxford, South Parks Road, Oxford OX1 3RE, UK

[5]Laboratory for Disease Mechanisms in Cancer, Department of Oncology, KU Leuven and Leuven Cancer Institute (LKI), Herestraat 49, 3000 Leuven, Belgium

[6]St James' Institute of Oncology, St James' University Hospital, Leeds LS9 7TF, UK

[7]Department of Oncology, Old Road Campus Research Building, University of Oxford, Oxford OX3 7DQ, UK

[8]Department of Haematology, Oxford University Hospitals NHS Trust, Oxford OX3 7JL, UK

[9]Lead Contact

*Correspondence: jan.rehwinkel@imm.ox.ac.uk

https://doi.org/10.1016/j.celrep.2020.107640

SUMMARY

The anti-leukemia agent forodesine causes cytotoxic overload of intracellular deoxyguanosine triphosphate (dGTP) but is efficacious only in a subset of patients. We report that SAMHD1, a phosphohydrolase degrading deoxyribonucleoside triphosphate (dNTP), protects cells against the effects of dNTP imbalances. SAMHD1-deficient cells induce intrinsic apoptosis upon provision of deoxyribonucleosides, particularly deoxyguanosine (dG). Moreover, dG and forodesine act synergistically to kill cells lacking SAMHD1. Using mass cytometry, we find that these compounds kill SAMHD1-deficient malignant cells in patients with chronic lymphocytic leukemia (CLL). Normal cells and CLL cells from patients without *SAMHD1* mutation are unaffected. We therefore propose to use forodesine as a precision medicine for leukemia, stratifying patients by *SAMHD1* genotype or expression.

INTRODUCTION

Intracellular deoxyribonucleoside triphosphate (dNTP) concentrations are controlled by dNTP synthesis and degradation. dNTPs are supplied by two pathways known as *de novo* and salvage. In the *de novo* pathway, dNTPs are synthesized from intracellular precursors. The enzyme ribonucleotide reductase catalyzes the rate-limiting step and converts ribonucleoside diphosphates into deoxyribonucleoside (dN) diphosphates (Hofer et al., 2012). The salvage pathway involves uptake of dNs from the extracellular environment, followed by intracellular phosphorylation by cytosolic and mitochondrial kinases to form dNTPs (Eriksson et al., 2002; Inoue, 2017; Reichard, 1988).

One enzyme that degrades intracellular dNTPs is the phosphohydrolase SAMHD1, initially identified as an interferon γ-inducible transcript in dendritic cells (Li et al., 2000). SAMHD1 cleaves all four dNTPs into the corresponding dNs and inorganic triphosphate (Goldstone et al., 2011; Powell et al., 2011). The catalytically active form of the protein is a homo-tetramer, the formation of which is regulated allosterically by dNTPs and guanosine triphosphate (GTP) as well as by phosphorylation of threonine 592 (reviewed in Ahn, 2016; Ballana and Esté, 2015). SAMHD1 has been studied extensively in the context of human immunodeficiency virus (HIV) infection. By limiting the supply of dNTPs for the viral reverse transcriptase, SAMHD1 blocks HIV infection in certain cell types (Hrecka et al., 2011; Laguette et al., 2011; Lahouassa et al., 2012; Rehwinkel et al., 2013). *SAMHD1* mutations cause Aicardi-Goutières syndrome (AGS), a rare autoinflammatory disease characterized by chronic production of type I interferons, a family of cytokines typically upregulated only during acute virus infection (Crow and Manel, 2015; Rice et al., 2009). Furthermore, mutations in the *SAMHD1* gene have been found in several types of cancer, including colorectal cancer and leukemias (Clifford et al., 2014; Johansson et al., 2018; Landau et al., 2015; Rentoft et al., 2016; Schuh et al., 2012). It is possible that inactivation of SAMHD1 provides transformed cells with a growth advantage simply due to elevated dNTP levels. Alternatively, the role of SAMHD1 in cancer may

This is an open access article under the CC BY license (http://creativecommons.org/licenses/by/4.0/).

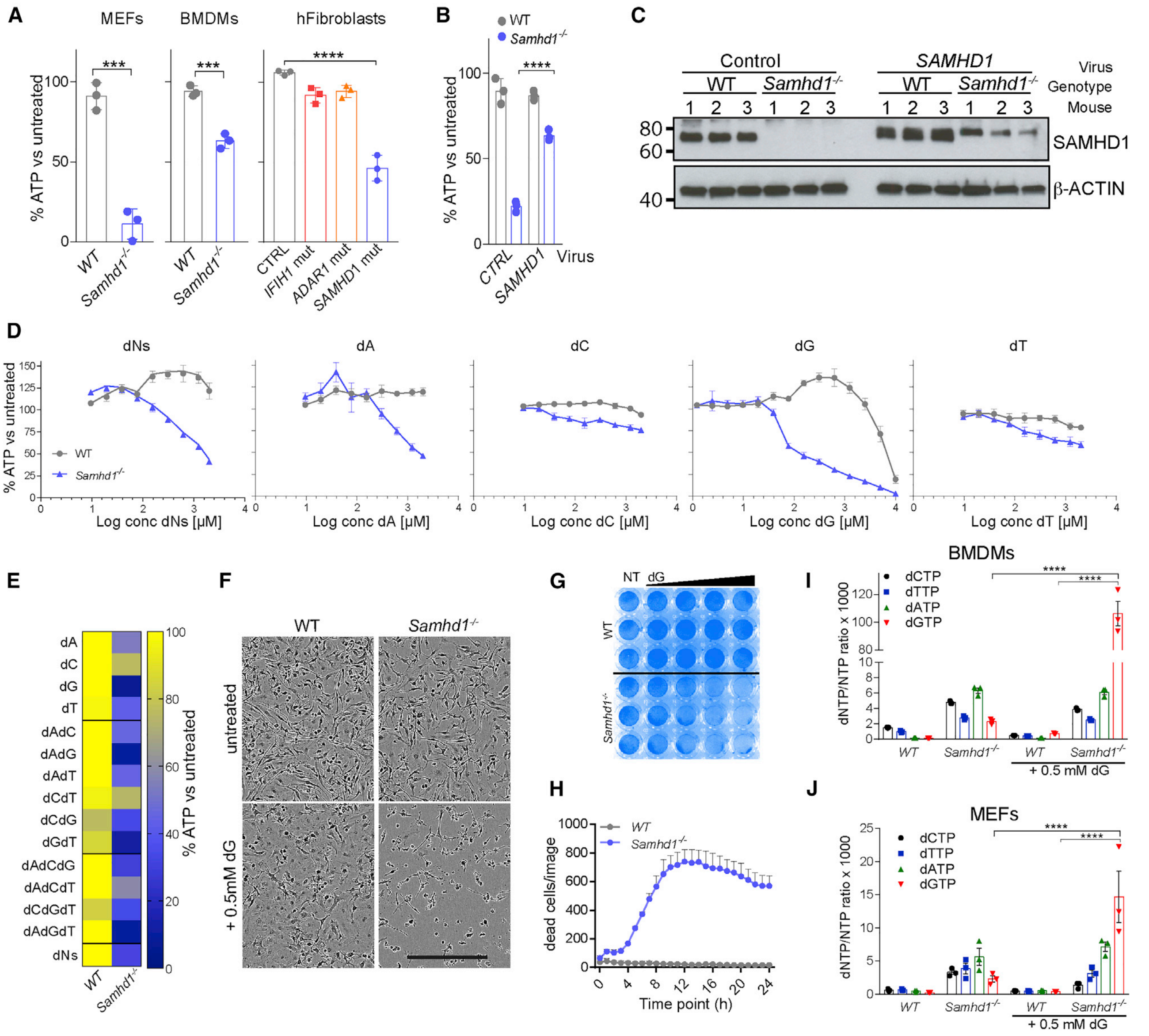

Figure 1. Deoxyribonucleosides (dNs) Are Toxic in SAMHD1-Deficient Cells

(A) Mouse embryonic fibroblasts (MEFs), BMDMs, and AGS patient-derived fibroblasts were treated with a mix of all four dNs. MEFs were cultured with 0.8 mM of each dN for 48 h. BMDMs and fibroblasts were treated with 0.5 mM of each dN for 24 h. Cell viability was determined by CellTiter-Glo assay. For each genotype, values from untreated control cells were set to 100%. Data from triplicate measurements are shown with mean ± SD. p values determined with unpaired t tests (MEFs and BMDMs) or one-way ANOVA (fibroblasts) are indicated.

(B and C) SAMHD1 expression was reconstituted in *Samhd1*$^{-/-}$ BMDMs. Cells of the indicated genotype were infected with a retrovirus expressing SAMHD1 or empty control retrovirus. Cells were then treated with 0.5 mM of each dN for 24 h. (B) Cell viability was tested as in (A). Values from triplicate measurements are shown with mean ± SD. p values determined with two-way ANOVA are indicated. (C) SAMHD1 expression was tested by western blot in BMDMs from three mice per genotype. β-Actin served as a loading control.

(D) BMDMs were treated with equimolar concentrations of all four dNs or with individual dNs at the indicated concentrations for 24 h. Cell viability was tested as in (A). Data from biological triplicates are shown as mean ± SEM.

(E) BMDMs were treated with individual dNs and combinations of dNs. Cells were cultured with 0.5 mM of the indicated dN(s) for 24 h, and viability was analyzed as in (A). Data from biological triplicates were averaged and are represented as a heatmap.

(F) BMDMs were treated with 0.5 mM dG for 24 h. Brightfield images are shown. Scale bar represents 300 µm.

(G) BMDMs were treated with increasing doses of dG for 24 h and fixed and stained with crystal violet. The wedge denotes 0.2, 0.4, 0.8, and 1.6 mM dG; NT, not treated.

(legend continued on next page)

relate to its functions in DNA repair and DNA replication, which are independent of dNTP degradation (Clifford et al., 2014; Coquel et al., 2018; Daddacha et al., 2017).

Chronic lymphocytic leukemia (CLL) is a very common form of adult leukemia and affects the elderly (Swerdlow, 2008). Refractoriness to chemotherapy and relapse remain major causes of death for patients with CLL. Nucleotide metabolism is an attractive target for the treatment of CLL and other leukemias. The small molecule forodesine (also known as Immucillin H or BCX-1777) was developed to inhibit purine nucleoside phosphorylase (PNP) (Kicska et al., 2001). PNP degrades deoxyguanosine (dG) into guanine, which is further catabolized into uric acid, which is released by cells (Gabrio et al., 1956). dG has cytotoxic properties (Dahbo and Eriksson, 1985; Mann and Fox, 1986; Theiss et al., 1976), and genetic PNP deficiency causes immunodeficiency and results in the loss of T cells and, in some patients, also affects B cell function (Markert, 1991). Upon forodesine treatment, dG accumulates intracellularly and is phosphorylated to deoxyguanosine triphosphate (dGTP). The resulting imbalance in dNTP pools is predicted to cause cell death and eliminate leukemic cells (Bantia et al., 2001). Furthermore, the synergy between dG and forodesine in inducing cell death *in vitro* has been suggested (Bantia et al., 2003), and, in patients, forodesine treatment increases plasma dG levels (Balakrishnan et al., 2006, 2010). Forodesine showed promising results *in vitro* in killing CLL B cells; surprisingly, however, it had substantial activity only in a small subset of patients with B or T cell malignancies (Alonso et al., 2009; Balakrishnan et al., 2006, 2010; Dummer et al., 2014; Gandhi and Balakrishnan, 2007; Gandhi et al., 2005; Maruyama et al., 2019).

Here, we explore the role of SAMHD1 in dNTP metabolism. We report that SAMHD1 protected cells against imbalances in dNTP pools. In cells lacking SAMHD1, engagement of the salvage pathway resulted in programmed cell death. Exposure to dG was particularly potent at inducing intrinsic apoptosis in SAMHD1-deficient primary and transformed cells. We further show that forodesine and other PNP inhibitors acted synergistically with dG to induce death in cells lacking SAMHD1. Importantly, *SAMHD1*-mutated leukemic cells without SAMHD1 expression from patients with CLL were selectively killed by forodesine and dG. This showed that SAMHD1 was limiting the potency of forodesine. It may therefore be possible to stratify patients with leukemia for forodesine treatment by *SAMHD1* genotype or expression.

RESULTS

SAMHD1 Protects Cells against dNTP Overload

To investigate the role of SAMHD1 in dNTP metabolism, we added equimolar concentrations of dNs to wild-type (WT) or SAMHD1-deficient cells. Surprisingly, widespread cell death was apparent by brightfield microscopy in cells lacking SAMHD1, but not in control cells after overnight incubation with dNs (data not shown). To study this phenotype systematically, we analyzed mouse embryonic fibroblasts (MEFs), mouse bone-marrow-derived macrophages (BMDMs), and primary human fibroblasts. Cell viability was assessed using a luminescence-based assay for intracellular ATP levels (CellTiter-Glo). We observed reduced viability of dN-exposed *Samhd1*$^{-/-}$ MEFs and BMDMs and human fibroblasts from a patient with AGS homozygously carrying the Q149X nonsense mutation in *SAMHD1* (Figure 1A). The viability of WT mouse and control human cells, including fibroblasts from patients with AGS carrying other AGS-causing mutations in *IFIH1* or *ADAR1*, was largely unaltered after the addition of dNs. To confirm that the absence of SAMHD1 renders cells susceptible to dN-induced cell death, we reconstituted BMDMs with a retrovirus expressing mouse SAMHD1. Indeed, expression of SAMHD1 in *Samhd1*$^{-/-}$ cells rescued viability after treatment with dNs (Figures 1B and 1C).

Next, we exposed BMDMs to increasing concentrations of dNs. We observed dose-dependent toxicity in *Samhd1*$^{-/-}$ cells, but not in WT cells, starting at ~0.1 mM dNs (Figure 1D). To determine if this effect was due to a specific dN, we treated BMDMs with single dNs. Interestingly, the highest toxicity in *Samhd1*$^{-/-}$ cells was observed when dG was used (Figure 1D). Like dG treatment, deoxyadenonsine (dA) also reduced viability specifically in SAMHD1-deficient cells, but at higher doses: a 50% reduction in intracellular ATP levels was observed with ~0.1 mM dG and ~1 mM dA (Figure 1D). Of note, dG also caused toxicity in WT cells at high doses above 5 mM (Figure 1D). We also tested dN combinations using a fixed dose of 0.5 mM. dG was the most toxic dN in *Samhd1*$^{-/-}$ cells when used alone or in combination with dA and/or thymidine, while the presence of deoxycytidine (dC) reduced the effect of dG on cell viability (Figure 1E). We therefore focused on dG in subsequent experiments at doses that did not reduce viability in WT cells. Brightfield images, crystal violet staining, and live-cell imaging confirmed the toxicity of dG in *Samhd1*$^{-/-}$ cells (Figures 1F–1H). In line with earlier work (Behrendt et al., 2013; Rehwinkel et al., 2013), the measurement of intracellular dNTP concentrations showed that the levels of all four dNTPs were elevated in *Samhd1*$^{-/-}$ cells (Figures 1I and 1J). Importantly, dG treatment resulted in 46-fold and 6-fold increases in dGTP concentrations in *Samhd1*$^{-/-}$ BMDMs and MEFs, respectively, while dGTP levels stayed largely unchanged in WT cells (Figures 1I and 1J). Taken together, these data show that exposure to dG led to dGTP accumulation in cells lacking SAMHD1, subsequently resulting in cell death.

dG Treatment Induces Apoptosis in *Samhd1*$^{-/-}$ Cells

We next determined the type of cell death triggered by dNs in *Samhd1*$^{-/-}$ cells. Annexin V and 7-aminoactinomycin D (7AAD) staining showed an increased frequency of early apoptotic (AnnexinV^{+}7AAD^{-}) and dead (AnnexinV^{+}7AAD^{+}) cells in dN-treated *Samhd1*$^{-/-}$ BMDM cultures (Figure 2A). Using the

(H) BMDMs were treated with 0.4 mM dG. Viability was monitored with the cell-impermeable dye Yoyo3 for 24 h using an in-incubator imaging system (Incucyte). Yoyo3^{+} cells were enumerated. Mean values from triplicate measurements are shown ± SD.
(I and J) BMDMs (I) and MEFs (J) were treated with 0.5 mM dG for 2 h, and intracellular dNTP levels were quantified relative to NTP levels. Data from three biological replicates are shown together with mean ± SEM. The p values determined with two-way ANOVA are indicated.
(A)–(C) and (F)–(H) are representative of at least three independent experiments. ***$p < 0.001$; ****$p < 0.0001$.

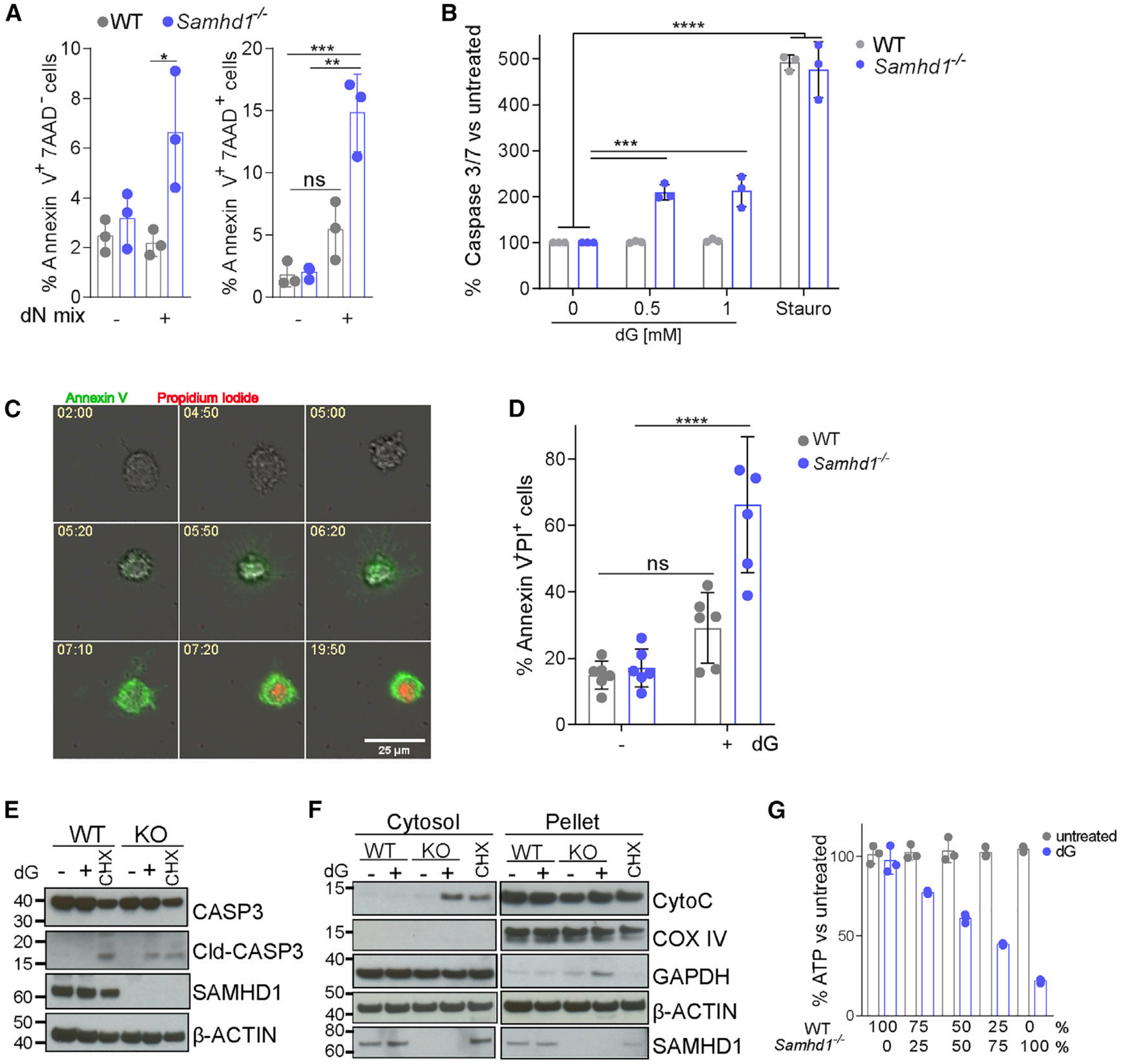

Figure 2. dG Treatment Kills *Samhd1*$^{-/-}$ Cells by Apoptosis

(A) BMDMs were treated with 0.5 mM of each dN for 24 h and stained with Annexin V and 7AAD. AnnexinV^{+}7AAD^{-} and AnnexinV^{+}7AAD^{+} cells were quantified by flow cytometry. Data from triplicate measurements are shown with mean ± SD. p values determined with two-way ANOVA are indicated.

(B) Caspase activity was assessed in BMDMs 6 h after treatment with the indicated doses of dG, or Staurosporine as control, using the Caspase 3/7 Glo assay. For each genotype, values from untreated control cells were set to 100. Data from triplicate measurements are shown with mean ± SD. The p value determined with an unpaired t test is indicated.

(C and D) Live-cell imaging of *Samhd1*$^{-/-}$ BMDMs treated with 0.5 mM dG. Alexa 488-labeled Annexin V and propidium iodide (PI) were added to the culture medium to visualize early apoptotic cells and cells that lost membrane integrity, respectively.

(C) Representative images of a *Samhd1*$^{-/-}$ cell treated with dG. Numbers show the time after dG exposure (h:min).

(D) Enumeration of AnnexinV^{+} PI^{+} cells after 24 h of treatment with or without 0.5 mM dG. Six images per condition were analyzed, and means ± SEM are shown. The p value determined with an unpaired t test is indicated.

(E and F) BMDMs were treated with 0.5 mM dG or 1 µg/mL cycloheximide (CHX, added to WT cells in F) for 8 hours.

(E) Levels of the indicated proteins in total cell extracts were determined by western blot.

(F) Cells were fractionated into cytosol and a pellet containing organelles. Levels of the indicated proteins were determined by western blot. β-Actin served as a loading control.

(G) WT and *Samhd1*$^{-/-}$ BMDMs were co-cultured at the indicated ratios. Cell viability was determined as in Figure 1A 24 h after treatment with 0.5 mM dG. Data from triplicate measurements are shown with mean ± SD.

(A)–(G) are representative of at least three independent experiments. ns, $p \geq 0.05$; *$p < 0.05$; **$p < 0.01$; ***$p < 0.001$.

See also Figure S1.

Caspase-Glo assay to measure the activity of apoptotic caspases, we found that the addition of dG to *Samhd1*$^{-/-}$ BMDMs, but not to WT cells, activated caspase 3/7 (Figure 2B). Live-cell imaging revealed that *Samhd1*$^{-/-}$ BMDMs treated with dG stained positive for Annexin V around 5 h post-treatment and subsequently for propidium iodide (PI) (Figure 2C). These observations suggest that dG treatment induced apoptosis, followed by secondary necrosis, rendering cells permeable to PI. Quantification of AnnexinV$^+$PI$^+$ cells by microscopy 24 h after dG exposure affirmed increased levels of dead *Samhd1*$^{-/-}$ BMDMs (Figure 2D). Cleaved caspase 3 was detectable by western blot in *Samhd1*$^{-/-}$, but not WT, BMDMs, supporting the notion that dG induced apoptosis (Figure 2E). Cycloheximide (CHX) served as a control in these experiments and induced caspase 3 cleavage in WT and *Samhd1*$^{-/-}$ cells. Cytochrome C is normally present in mitochondria and released into the cytosol early during intrinsic apoptosis. To characterize the apoptosis pathway activated by dG treatment, we measured cytochrome C levels in the cytosol and found that treatment of *Samhd1*$^{-/-}$ BMDMs with dG led to a redistribution of cytochrome C into the cytosol (Figure 2F). To investigate whether a soluble factor was triggering apoptosis, we co-cultured WT and *Samhd1*$^{-/-}$ BMDMs and treated them with dG. Viability decreased with increasing proportions of *Samhd1*$^{-/-}$ cells present in the co-culture, suggesting that apoptosis was induced in a cell-autonomous fashion (Figure 2G). Altogether, these results show that dG triggered intrinsic apoptosis in *Samhd1*$^{-/-}$ cells.

Nuclear DNA Replication Is Not Required for dG-Induced Apoptosis

Earlier work in yeast showed that severe dNTP pool imbalances can trigger stalled replication forks and checkpoint activation (Kumar et al., 2011; Poli et al., 2012). To study the role of DNA replication in dG-induced death of SAMHD1-deficient cells, we analyzed cell cycle progression in BMDMs by BrdU and PI staining after dG treatment. Untreated WT and *Samhd1*$^{-/-}$ BMDM cultures contained ~20% BrdU$^+$ cells, indicative of cells in S-phase with ongoing DNA replication (Figure S1A). After dG treatment, WT cells already in S-phase progressed through the cell cycle. At the same time, new cells did not enter S-phase, resulting in a much reduced population of BrdU$^+$ cells after 24 h of dG exposure. In contrast, *Samhd1*$^{-/-}$ cells in S-phase did not progress. Instead, a population of cells displaying sub-G0/G1 PI staining, indicative of dead cells that lost their nucleic acid content, was detected in *Samhd1*$^{-/-}$ cultures, starting at 8 h after dG treatment (Figure S1A). Next, we performed a BrdU pulse-chase experiment in which we labeled BMDMs with BrdU first and then treated with dG. Over time, WT cultures accumulated a distinct population of G0/G1-BrdU$^+$ cells and contained fewer cells in S-phase (Figure S1B). This confirmed that WT cells progressed through the cell cycle but did not enter S-phase. In *Samhd1*$^{-/-}$ cultures, cells with sub-G0/G1 PI staining were evident from 8 h onward. These included both BrdU$^+$ and BrdU$^-$ cells, suggesting that dG treatment killed both cycling and non-cycling *Samhd1*$^{-/-}$ cells (Figure S1B). We therefore tested whether DNA replication was required for the toxicity of dG in *Samhd1*$^{-/-}$ cells. BMDMs were cultured in serum-free medium (R0) or pre-treated with hydroxyurea (HU), both of which induced cell cycle arrest, evident from reduced numbers of cells in S-phase (Figure S1C). *Samhd1*$^{-/-}$ cells arrested by both methods were susceptible to killing by dG (Figures S1D and S1E). We also pre-treated BMDMs with aphidicolin (APD), which blocks nuclear, but not mitochondrial, DNA polymerases (Lentz et al., 2010; Zimmermann et al., 1980). As expected, APD-treated cells were arrested in early S-phase (Figure S1F). Interestingly, APD-treated *Samhd1*$^{-/-}$ cells were susceptible to dG-induced toxicity (Figure S1G). Finally, we assessed oxidative stress by measuring levels of the reactive oxygen species H_2O_2 and found that dG treatment of WT and SAMHD1-deficient BMDMs did not induce oxidative stress (Figure S1H). As a control, we used menadione that induced equivalent H_2O_2 levels in cells irrespective of their genotype. Together, these data suggest that dG-induced apoptosis occurred independently of nuclear DNA replication and oxidative stress and that dGTP overload was toxic in both cycling and non-cycling *Samhd1*$^{-/-}$ cells.

dG Treatment Kills SAMHD1-Deficient Cancer Cells

SAMHD1 mutations are present in several types of cancer and, in many cases, result in reduced mRNA and protein levels (Clifford et al., 2014; Johansson et al., 2018; Rentoft et al., 2016). We therefore wished to explore our finding of dN-induced cell death in the context of malignant disease. Initially, we tested cancer cell lines. Vpx is a HIV-2 accessory protein that targets SAMHD1 for proteasomal degradation (Hrecka et al., 2011; Laguette et al., 2011). We used virus-like particles (VLPs) containing Vpx to deplete SAMHD1 in the cervical cancer cell line HeLa and the breast cancer cell line MDA-MB231, which both express SAMHD1. Cells treated with VLP_{vpx}, but not with control VLPs lacking Vpx (VLP_{ctrl}), showed reduced viability upon addition of dNs or dG (Figures 3A–3D). SAMHD1 staining and analysis by flow cytometry or SAMHD1 western blot showed SAMHD1 depletion using VLP_{vpx} in HeLa and MDA-MB231 cells, respectively (Figures S2A–S2C). In addition, we generated a *Samhd1*$^{-/-}$ B16F10 mouse melanoma cell line using CRISPR-Cas9 (strategy and validation shown in Figures S2D and S2E). *Samhd1*$^{-/-}$ B16F10 cells showed increased frequencies of early apoptotic (AnnexinV$^+$7AAD$^-$) and dead (AnnexinV$^+$7AAD$^+$) cells upon dG treatment, accompanied by reduced confluency (Figures 3E–3G and S2F). We made similar observations in the murine colorectal cancer cell line CT26 upon SAMHD1 knockout (data not shown). We also included Jurkat cells in our analysis, a human T cell line that, in contrast to the other cell lines utilized, does not express SAMHD1 (Baldauf et al., 2012). Jurkat cells were exquisitely sensitive to dG treatment at doses approximately 10 times lower than those used in most other experiments (Figure 3H). We confirmed the killing of Jurkat cells by testing their clonogenic potential, which was greatly reduced upon dG treatment (Figure 3I). Reconstitution with a lentivirus expressing human SAMHD1 partially rescued viability upon dG treatment (Figures 3J and 3K). Interestingly, SAMHD1 K11A, which does not localize to the cell nucleus but remains active as a dNTPase (Schaller et al., 2014), executed an even more pronounced rescue as compared to WT SAMHD1 (Figures 3J and 3K). In contrast, SAMHD1 H233A, which lacks catalytic activity, was largely defective in rescuing viability upon dG treatment

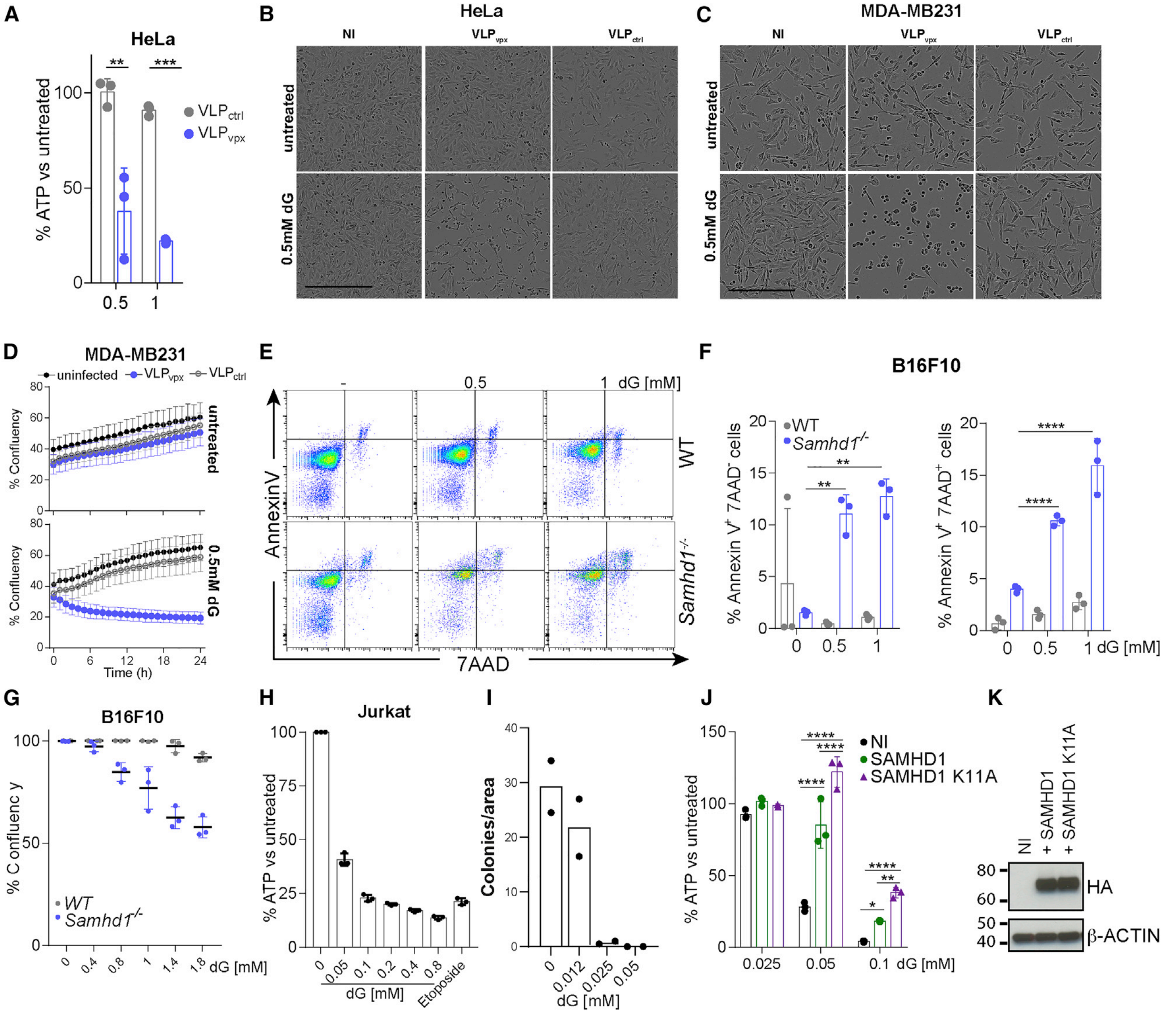

Figure 3. dG Induces the Death of Cancer Cell Lines

(A) HeLa cells were infected with VLPs containing Vpx (VLP_{vpx}) or not (VLP_{ctrl}). After 24 h, cells were treated with 0.5 mM of each dN for an additional 24 h. Cell viability was assessed as in Figure 1A.

(B and C) HeLa (B) and MDA-MB231 (C) cells were left uninfected (NI) or were infected with VLPs containing Vpx (VLP_{vpx}) or not (VLP_{ctrl}). After 24 h, cells were treated with 0.5 mM dG, and brightfield images were acquired after an additional 10–12 h. Scale bars represent 300 μm.

(D) MDA-MB231 cells were treated as in (C), and confluency was monitored after dG addition using a live-cell imaging system in the incubator (Incucyte). The mean of 9 measurements ± SD is shown.

(E–G) Wild-type and *Samhd1*$^{-/-}$ B16F10 cells were treated with dG as indicated for 20 h.

(E and F) Cells were then stained with Annexin V and 7AAD and analyzed by flow cytometry. Representative fluorescence-activated cell sorting (FACS) plots are shown in (E), and Annexin V^+ $7AAD^-$ and Annexin V^+ $7AAD^+$ cells were quantified in (F).

(G) Confluency was determined as in (D).

(H) Jurkat cells were treated for 20 h with dG as indicated or with 25 μM etoposide. Cell viability was determined as in Figure 1A.

(I) Jurkat cells were treated with dG as indicated for 20 h and then seeded in semi-solid medium containing dG. After 13 days, cell colonies were counted, and the number colonies per field of view are shown.

(J and K) Jurkat cells were reconstituted with hemagglutinin (HA)-tagged wild-type or K11A mutant SAMHD1 using a lentivector. Uninfected cells (NI) served as control.

(J) Cells were then treated with dG for 48 h. Cell viability was determined as in Figure 1A.

(K) SAMHD1 levels in total cell extracts were determined by western blot. β-Actin served as a loading control.

(A), (D)–(H), and (J)–(K) are representative of three independent experiments and (B) and (C) of two experiments. In (A), (F)–(H), and (J), dots represent technical triplicates and means ± SD are shown. In (I), data from two independent experiments were pooled, and dots represent the mean of technical duplicates per experiment. The p values determined with two-way ANOVA are indicated. $^{*}p < 0.05$; $^{**}p < 0.01$; $^{***}p < 0.001$; $^{****}p < 0.0001$.

See also Figures S2 and 3.

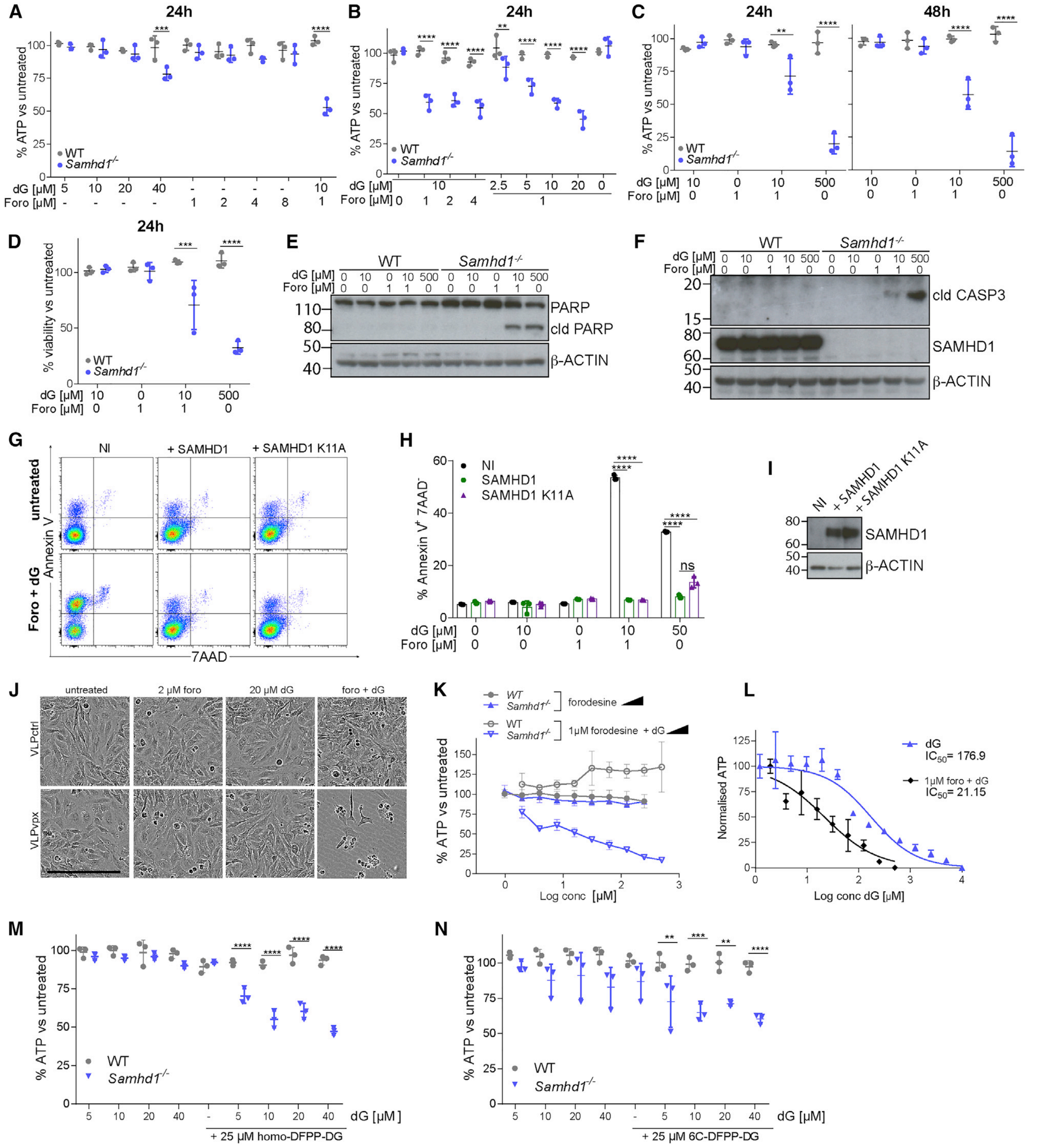

Figure 4. PNP Inhibitors and dG Synergistically Induce Cell Death in Cells Lacking SAMHD1

(A–C) BMDMs were treated with the indicated doses of dG and forodesine. Viability was tested as in Figure 1A after 24 or 48 h.

(D) BMDMs treated for 24 h with dG and forodesine were fixed and stained with crystal violet. After washing, cell-associated dye was solubilized and quantified by absorbance at 570 nm. For each genotype, values from untreated control cells were set to 100%.

(E and F) BMDMs were treated for 8 h with dG and forodesine. Levels of PARP and cleaved PARP (E) or cleaved CASPASE 3 and SAMHD1 (F) in total cell extracts were determined by western blot. β-Actin served as a loading control. cld, cleaved.

(G–I) Jurkat cells were reconstituted with SAMHD1 as described in Figures 3J and 3K. Uninfected cells (NI) served as control.

(G and H) Cells were treated for 18 h with 10 μM dG and 1 μM forodesine. Cells were then stained with Annexin V and 7AAD and analyzed by flow cytometry. Representative FACS plots are shown in (G) and Annexin V^{+} 7AAD^{-} cells are quantified in (H).

(legend continued on next page)

(Figure S3). In conclusion, SAMHD1 protected cancer cell lines against dN-triggered toxicity.

SAMHD1 Protects against Combined Forodesine and dG Treatment

Forodesine is an inhibitor of PNP, which converts dG into guanine and α-D-ribose 1-phosphate, and has been shown to induce apoptosis in leukemic cells, possibly by increasing the intracellular and plasma concentrations of dG and consequently intracellular dGTP (Balakrishnan et al., 2010; Bantia et al., 2010; Kicska et al., 2001; Posmantur et al., 1997). Our system, in which SAMHD1-deficient cells fed with dG died by apoptosis due to dGTP overload, resembled this situation. We therefore hypothesized that forodesine and dG might work synergistically in SAMHD1-deficient cells. Indeed, low doses of dG or forodesine alone did not compromise the viability of WT or *Samhd1*$^{-/-}$ BMDMs, while the combination of both was toxic in *Samhd1*$^{-/-}$ cells (Figures 4A, and 4B). We confirmed this observation using 10 μM dG and 1 μM forodesine at (1) different time points, (2) with crystal violet staining, and (3) biochemically by cleavage of poly (ADP-ribose) polymerase (PARP) and CASPASE3 (Figures 4C–4F). Jurkat cells treated with the same doses of forodesine and dG showed increased proportions of AnnexinV$^+$7AAD$^-$ cells, and this was prevented when SAMHD1 or SAMHD1 K11A were expressed (Figures 4G–4I). On the other hand, SAMHD1-sufficient HeLa cells were sensitized to combined forodesine and dG treatment by Vpx-mediated depletion of SAMHD1 (Figure 4J).

To further study the synergy between forodesine and dG in cells lacking SAMHD1, we titrated forodesine and dG. Forodesine alone had no effect on the viability of *Samhd1*$^{-/-}$ BMDMs, including at high doses (Figure 4K, closed symbols). In contrast, dG triggered dose-dependent toxicity in the presence of 1 μM forodesine in *Samhd1*$^{-/-}$ cells, but not in WT cells (Figure 4K, open symbols). Comparing the dose response to dG in the presence or absence of forodesine revealed that forodesine sensitized SAMHD1-deficient BMDMs to dG by approximately 10-fold (Figure 4L).

Finally, we tested whether other PNP inhibitors might induce death of SAMHD1-deficient cells in the presence of dG. Indeed, BMDMs showed reduced viability upon exposure to either homo-DFPP-DG or 6C-DFPP-DG (Glavas-Obrovac et al., 2010; Hikishima et al., 2007, 2010) together with low doses of dG (Figures 4M and 4N). Taken together, these data show that SAMHD1 protected cells against death that was synergistically induced by PNP inhibitors and dG. Thus, our observations reveal a key role of SAMHD1 in the mechanism underlying the toxicity of compounds such as forodesine.

CLL B Cells with *SAMHD1* Mutations Are Highly Sensitive to a Combination Treatment of Forodesine and dG

SAMHD1 is mutated in 11% of patients with refractory CLL (Clifford et al., 2014). Since SAMHD1 protected cells against treatment with forodesine and dG (Figure 4), we hypothesized that CLL B cells with *SAMHD1* mutations would be particularly susceptible to this combination treatment. To test this, we compared the effect of forodesine and dG treatment on peripheral blood mononuclear cells (PBMCs) from patients with CLL with or without acquired mutations in *SAMHD1*.

Details of the genetic status and *SAMHD1* mutations of the patients' cells are shown in Figures S4A and S4B. PBMCs from patients with CLL were treated with 2 μM forodesine, 20 μM dG, or both. When used alone, neither forodesine nor dG significantly reduced cell viability assessed by intracellular ATP content (Figure 5A). The combination of both compounds had little effect on the viability of PBMCs from patients without *SAMHD1* mutations. However, significantly reduced PBMC viability was observed in the *SAMHD1*-mutated group (Figure 5A). These data were confirmed by flow cytometry: the population of live cells was selectively reduced after forodesine and dG treatment of PBMCs from patients with *SAMHD1* mutations (data not shown).

To investigate which types of cells were affected by exposure to forodesine and dG, we used cytometry by time of flight (CyTOF) analysis. PBMCs from healthy control subjects and patients with CLL were treated or not with both compounds. After treatment, cells were stained with a panel of antibodies recognizing cell surface markers to identify cell types. SAMHD1 expression, phosphorylation of nuclear factor κB (NF-κB)-p65, p38, and STAT1, and cleavage of PARP and CASPASE3 were also monitored by intracellular staining. CLL B cells were marked by co-expression of CD5 and CD19 (Swerdlow, 2008). As expected, CD5$^+$CD19$^+$ cells (CLL B cells) were largely absent from control PBMCs and could be detected at varying frequencies in samples from patients with CLL, irrespective of *SAMHD1* genotype (Figures 5B, 5C, S4C, and S4D). We also analyzed SAMHD1 expression in CLL B cells. Variable expression of SAMHD1 was observed in the *SAMHD1* non-mutated group, while CLL B cells from the *SAMHD1*-mutated group had no detectable levels of SAMHD1 (Figures 5D and S4C). This shows that the *SAMHD1* mutations studied here resulted in a loss of SAMHD1 protein, in line with our earlier observations (Clifford et al., 2014).

Next, we analyzed our CyTOF data using viSNE (Amir et al., 2013; Kimball et al., 2018). This analysis tool uses the t-Distributed Stochastic Neighbor Embedding (tSNE) algorithm and displays high-dimensional data on a two-dimensional map. Each

(I) SAMDH1 levels in total cell extracts were determined by western blot. β-Actin served as a loading control.
(J) HeLa cells were infected with VLPs containing Vpx (VLP_{vpx}) or not (VLP_{ctrl}). After 6 h, cells were treated with 20 μM dG and 2 μM forodesine, and brightfield images were acquired after an additional 48 h. Scale bar represents 300 μm.
(K) BMDMs were treated with the indicated doses of dG and forodesine. Viability was tested as in Figure 1A after 24 h. Means from three biological replicates are shown ± SEM.
(L) *Samhd1*$^{-/-}$ BMDMs were treated with the indicated doses of dG in the presence or absence of 1 μM forodesine. Cell viability was determined by CellTiter-Glo assay after 24 h. Data were normalized by setting the values for the lowest and highest dG concentrations to 100 and 0, respectively. Means from three biological replicates are shown ± SEM. Half maximal inhibitory concentration (IC_{50}) values were calculated from the non-linear regression curves shown on the graph.
(M and N) BMDMs were treated with the indicated doses of dG and homo-DFPP-DG (M) or 6C-DFPP-DG (N). Viability was tested as in Figure 1A after 24 h. Data are representative of three independent experiments. In (A)–(D), (M), (N), and (H), dots represent BMDMs from individual mice and technical replicates, respectively. Mean ± SD is shown. The p values determined with two-way ANOVA are indicated. $^{**}p < 0.01$; $^{***}p < 0.001$; $^{****}p < 0.0001$

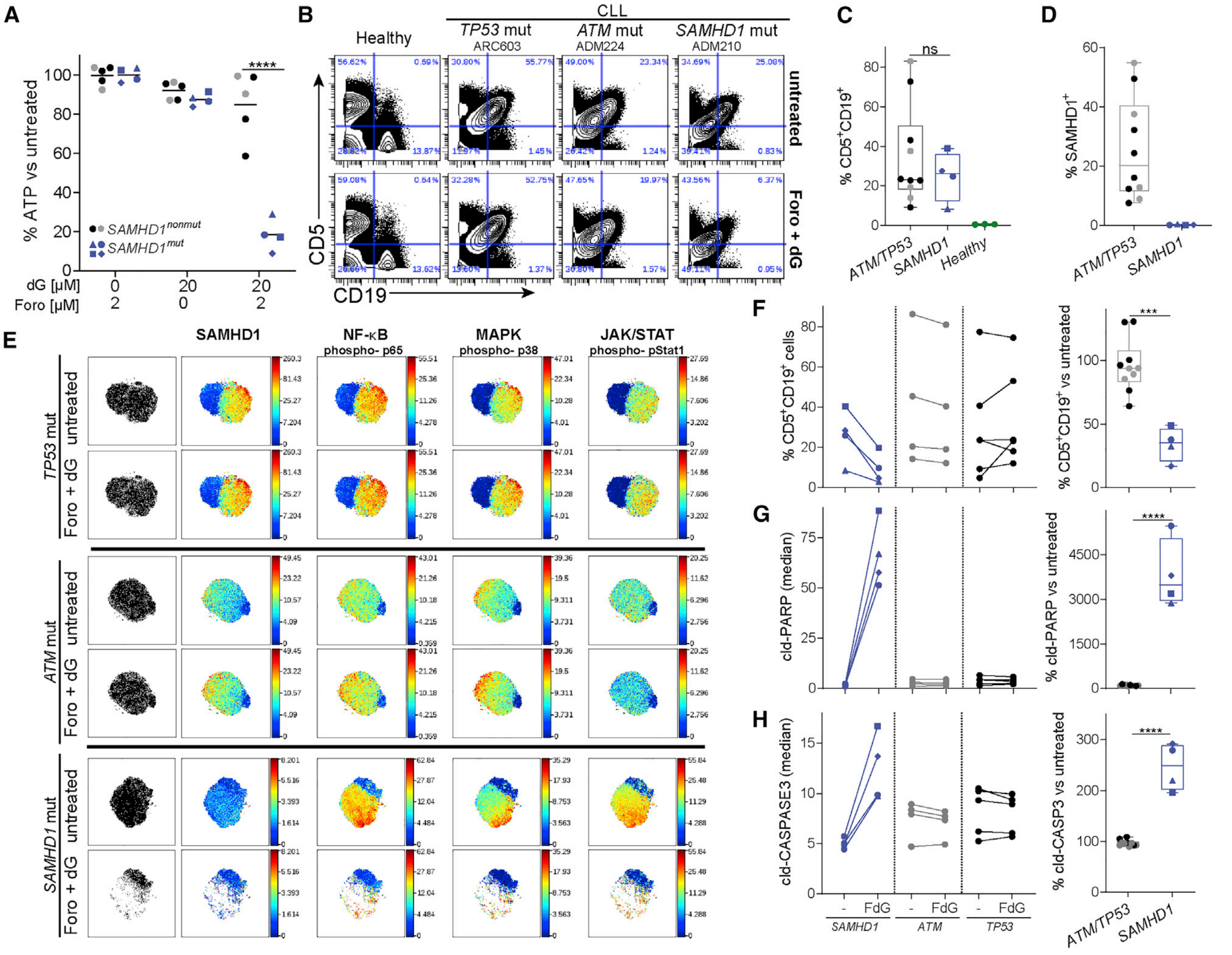

Figure 5. Elimination of *SAMHD1*-Mutated Leukemic Cells by Forodesine and dG Treatment

(A) PBMCs from patients with CLL were treated for 24 h with dG and forodesine as indicated. Viability was tested as in Figure 1A. Details on *SAMHD1* genetic status are provided in Figures S4A and 4B.

(B–H) PBMCs from healthy control subjects and patients with CLL were treated or not for 24 h with 20 µM dG and 2 µM forodesine (Foro + dG). Cells were then analyzed using CyTOF.

(B) Live cells were gated (see Figure S4C). The CD5 and CD19 staining is shown for selected samples (see Figure S4D for all samples).

(C) Percentages of untreated, live $CD5^{+}CD19^{+}$ cells are shown.

(D) SAMHD1 expression was analyzed in untreated, live $CD5^{+}CD19^{+}$ cells, and the percentage of $SAMHD1^{+}$ cells is shown (see Figure S4C for gating).

(E) Live $CD5^{+}CD19^{+}$ cells from each sample were analyzed separately by viSNE using 22 lineage markers (Cytobank; settings: 1,000 iterations, 30 perplexity, and 0.5 theta). Representative tSNE plots are shown (see Figure S5 for all samples) and were colored by expression or phosphorylation of the indicated markers.

(F) Left, percentages of $CD5^{+}CD19^{+}$ cells among all live cells are shown in untreated and treated PBMC samples. FdG, treatment with 2 µM forodesine and 20 µM dG. Right, the frequency of live $CD5^{+}CD19^{+}$ cells was set to 100 in untreated samples, and their percentage after forodesine and dG treatment is shown.

(G–H) The staining for cleaved PARP (G) and cleaved CASPASE3 (H) in live $CD5^{+}CD19^{+}$ cells was analyzed. Left, median values are shown in untreated and treated cells. Right, median values from untreated cells were set to 100 separately for each sample.

In (A), (C), (D), and (F)–(H), dots represent cells from different patients and the color indicates the mutation status (gray, *ATM* ; black, *TP53*; blue, *SAMHD1*). Horizontal bars represent means. In (C) and (D), box and whiskers show SD and maximum/minimum values, respectively. The p values determined with two-way ANOVA (A) or unpaired t test (C),(D), and (F)–(H) are indicated. ns, $p \geq 0.05$; *$p < 0.05$; **$p < 0.01$; ***$p < 0.001$; ****$p < 0.0001$.

See also Figures S4–S6.

dot on a viSNE plot corresponds to a cell. Color can be used to show the expression of a chosen parameter. We generated viSNE plots after gating on CLL B cells (Figures 5E and S5). tSNE maps showed a marked reduction of CLL B cells upon forodesine and dG treatment in cells from *SAMHD1*-mutated patients, but not in the *SAMHD1* WT groups (Figures 5E and S5). Gating on CLL B cells also confirmed that forodesine and dG treatment resulted in their selective loss in the *SAMHD1*-mutated group, while there was no effect on the same cell population in the *SAMHD1* non-mutated group (Figure 5F). Significantly increased levels of cleaved PARP and cleaved CASPASE3 were observed only in *SAMHD1*-mutated CLL B cells post-

treatment, consistent with the induction of apoptosis (Figures 5G and 5H).

Interestingly, forodesine and dG ablated a subpopulation of *SAMHD1*-mutated CLL B cells, which appeared to be characterized by high levels of NF-κB-p65, p38, and STAT1 phosphorylation (Figures 5E and S5). Using phosphorylated (p-)p38, a mitogen-activated protein (MAP) kinase, we defined "active" and "inactive" cells and analyzed the expression of selected markers in these subpopulations (Figure S6A). This analysis confirmed that p-p38-positive cells also displayed higher levels of p-p65 and p-STAT1 and revealed higher levels of CD27 expression in these "active" cells, which may indicate engagement of the B cell receptor (Lafarge et al., 2015). Interestingly, staining for cleaved PARP and cleaved CASPASE3 was enhanced more strongly in "inactive" cells upon treatment (Figures S6B and S6C). This suggests that these "inactive" CLL B cells were also affected by the treatment and induced apoptosis with delayed kinetics compared to "active" cells.

Collectively, these data show that forodesine and dG were highly efficient at killing malignant CLL B cells with *SAMHD1* mutations that cause a defect in SAMHD1 expression, while cells with intact SAMHD1 expression were spared.

DISCUSSION

Our data reveal an unexpected role of SAMHD1 in safeguarding cells against cell death resulting from imbalances in dNTP pools. In the absence of SAMHD1, dNTP imbalances induced by exposure of cells to dNs triggered apoptosis. This phenotype was observed in a wide range of human and mouse cells, including primary and transformed cells. We further show synergy between dG exposure and forodesine, which blocks dG degradation by PNP, in cells lacking SAMHD1. Importantly, the combination of dG and forodesine selectively killed SAMHD1-deficient CLL B cells, while other normal cells or SAMHD1-sufficient CLL B cells remained unaffected.

Since its identification as a restriction factor for HIV (Hrecka et al., 2011; Laguette et al., 2011), SAMHD1 has been studied extensively in the context of lentiviral infections. Interestingly, SAMHD1 is highly conserved from marine invertebrates to man (Rice et al., 2009), whereas lentiviruses evolved much more recently. This suggests that restriction of lentiviral infection is an exaptation of SAMHD1's biochemical activity to degrade dNTPs, which perhaps has a more ancestral function in cellular dNTP metabolism. We propose that this evolutionarily conserved function of SAMHD1 is to correct imbalances in dNTP pools, thereby safeguarding against cell death. Indeed, we report that intracellular dNTP concentrations were only marginally altered in WT cells exposed to extracellular dNs, while SAMHD1-deficient cells accumulated large dNTP pools. Concomitantly, cells lacking SAMHD1 succumbed to apoptotic cell death.

Interestingly, dG was the most toxic dN. This may be related to the observation that baseline dGTP concentrations are lower than those of the three other dNTPs, resulting in particularly pronounced dNTP imbalances upon dG feeding. In addition, dGTP allosterically regulates ribonucleotide reductase, preventing dCTP production through the *de novo* pathway (Moore and Hurlbert, 1966). Consistent with this idea is our observation that the toxicity of dG was reduced when added together with dC, providing dCTP via the salvage pathway. In addition, dC may indirectly increase dTTP concentrations via a pathway involving dCMP deaminase and thymidylate synthase (Theiss et al., 1976), thereby balancing dNTP levels.

The molecular mechanism by which dNTP imbalances cause cell death is a long-standing question (Gudas et al., 1978; Mann and Fox, 1986), and future work will be required to elucidate how apoptosis is triggered in SAMHD1-deficient cells containing elevated dGTP pools. We observed release of cytochrome C from mitochondria into the cytosol, indicative of cell-intrinsic apoptosis. This notion was supported by the observation that cell death in mixed cultures containing WT and *Samhd1*$^{-/-}$ cells was proportional to the fraction of knockout cells. We further found that dN-triggered cell death did not require ongoing nuclear DNA synthesis. It is therefore possible that dNTP imbalances disrupt replication or repair of mitochondrial DNA, resulting in mitochondrial stress and subsequent apoptosis (Arpaia et al., 2000; Franzolin et al., 2015). dGTP may also be involved more directly in the activation of apoptosis, as has been reported for dATP (Li et al., 1997; Reubold et al., 2009). Alternatively, dN treatment might have indirect effects on the induction of apoptosis in the absence of SAMHD1. Indeed, in THP1 cells, SAMHD1 knockout results in increased cell proliferation and altered cell cycle and apoptosis control (Bonifati et al., 2016).

Clinical trials showed that forodesine has beneficial effects in some, but not all, patients with B or T cell malignancies (Alonso et al., 2009; Balakrishnan et al., 2006, 2010, 2013; Dummer et al., 2014; Gandhi and Balakrishnan, 2007; Gandhi et al., 2005; Maruyama et al., 2019; Ogura et al., 2012), an observation that thus far has lacked an explanation. This study and our earlier work shows that *SAMHD1* mutations found in patients with CLL often result in the loss of expression at mRNA and protein levels (Clifford et al., 2014). Importantly, our data suggest that forodesine-sensitive leukemias harbor mutations that ablate SAMHD1 expression. It is possible that *SAMHD1* mutations found in some patients with CLL do not affect SAMHD1 protein levels. We speculate that such mutations will sensitize cells to forodesine if they impair SAMHD1's dNTPase activity. It may therefore be possible to stratify patients by *SAMHD1* genotype, expression levels, or protein function. *SAMHD1* mutations are found in 3%–5% of newly diagnosed CLL and expand in relapsed and refractory disease to a frequency of about 11% (Clifford et al., 2014; Landau et al., 2015). As such, only a subset of patients with CLL is likely to benefit from forodesine. However, CLL is the most common leukemia in the Western world; thus, significant numbers of patients with CLL have *SAMHD1* mutations. Furthermore, *SAMHD1*-mutated cases show poorer response to conventional first-line chemoimmunotherapy compared to *SAMHD1* WT patients. Thus, the results presented here are highly relevant and clinically significant to expand choices of first-line treatments to this specific patient group (Clifford et al., 2014).

Retrospective analysis of previous clinical trials with forodesine could lend support to the idea of stratifying patients by SAMHD1 status. However, restricted sample availability, limited consent to obtain genetic information, and small trial sizes have precluded this approach. Instead, future clinical trials should be conducted to determine whether the efficacy of forodesine can

CellPress
OPEN ACCESS

be predicted by the presence or absence of SAMHD1 in transformed cells. Survival of PBMCs *ex vivo* upon forodesine and dG treatment (Figure 5A) and commercially available α-SAMHD1 antibodies would be suitable for rapid clinical assays to identify patients with SAMHD1 deficiency. Previous studies reported that dG needs to be added in combination with forodesine to induce toxicity *in vitro* (Alonso et al., 2009; Balakrishnan et al., 2006; Bantia et al., 2001; Gandhi and Balakrishnan, 2007; Gandhi et al., 2005). In patients, forodesine treatment alone has been shown to increase plasma dG levels (Balakrishnan et al., 2006, 2010). However, it may also be interesting to explore supplementing forodesine with dG in patients with leukemia with acquired *SAMHD1* mutations. It is noteworthy that SAMHD1 is broadly expressed in most normal human tissues (Schmidt et al., 2015; Uhlén et al., 2015). As such, forodesine and dG are unlikely to have negative effects on healthy cells.

Our CyTOF analysis revealed that CLL B cells, identified by CD19 and CD5 staining, contained two subpopulations of cells, distinguishable by expression of CD27 and phosphorylation of p65, p38, and STAT1. Activation of NF-κB, MAP kinase, and STAT signaling pathways has been reported in CLL (Frank et al., 1997; Herishanu et al., 2011; Ringshausen et al., 2004; Shukla et al., 2018), and CD27 is upregulated in response to B cell receptor engagement (Lafarge et al., 2015). We therefore labeled these cell populations "active" and "inactive." Interestingly, we found that forodesine and dG not only killed the "active" population of CLL B cells, but also induced markers of apoptosis in the "inactive" cells. As such, forodesine may have an advantage over other CLL drugs that inhibit B cell receptor signaling and thus target only "active" cells.

In an independent line of investigations, SAMHD1 was found to not only degrade naturally occurring dNTPs, but also some nucleotide analogs, including cytarabine (ara-C) and decitabine (DAC), which are used for the treatment of acute myeloid leukemia (AML) (Herold et al., 2017a, 2017b; Hollenbaugh et al., 2017; Oellerich et al., 2019; Schneider et al., 2017). The response of patients with AML to ara-C or DAC inversely correlates with SAMHD1 expression levels or activity (Herold et al., 2017a; Oellerich et al., 2019; Rudd et al., 2020; Schneider et al., 2017). These observations are an interesting parallel to our work and highlight SAMHD1 as a target for cancer therapy.

In summary, we uncovered an important role of SAMHD1 in protecting cells against dNTP imbalance that otherwise triggers apoptotic cell death. These findings allowed us to selectively ablate *SAMHD1*-mutated transformed cells that lacked SAMHD1 expression using a combination treatment involving forodesine and dG. In the future, forodesine may be developed into a precision medicine for a subset of patients with leukemia with acquired *SAMHD1* mutations.

STAR★METHODS

Detailed methods are provided in the online version of this paper and include the following:

- KEY RESOURCES TABLE
- RESOURCE AVAILABILITY
  - Lead Contact
  - Materials Availability
  - Data and Code Availability
- EXPERIMENTAL MODEL AND SUBJECT DETAILS
  - Mice
  - Cells
  - Samples from Patients with CLL
  - Study Approval
- METHOD DETAILS
  - Plasmids
  - dNTP Measurements
  - Viability Assays
  - Apoptosis Assays
  - Cell Cycle Analysis
  - Clonogenic Assay
  - Measurement of ROS Production
  - Western Blots
  - Retroviral Vectors
  - Stimulation, Staining, and Mass Cytometry Analysis of Patient Samples
  - Generation of Samhd1$^{-/-}$ B16F10 Cells
- QUANTIFICATION AND STATISTICAL ANALYSIS

SUPPLEMENTAL INFORMATION

Supplemental Information can be found online at https://doi.org/10.1016/j.celrep.2020.107640.

ACKNOWLEDGMENTS

The authors thank Y. Crow and G.I. Rice for providing primary human fibroblasts, T. Yokomatsu for homo-DFPP-DG and 6C-DFPP-DG, and T. Schaller for SAMHD1 expression vectors. We thank Q. Sattentau, members of the Rehwinkel lab, J. Maelfait, P. Borrow, J. McKeating, P. Pasero, Y.L. Lin, S. Kriaucionis, W. Niedzwiedz, and V. Cerundolo for discussion. We acknowledge G. Napolitani and M. Mazurczyk for their help in the mass cytometry facility at the WIMM for providing technical expertise, cell analysis services, and scientific input. The facility is supported by the MRC HIU core-funded project (MC_UU_00008) and the Oxford Single Cell Biology Consortium (OSCBC). We thank P, Hublitz for his help with the generation of *Samhd1*$^{-/-}$ B16F10 cells. The WIMM Genome Engineering Facility is supported by grants from the MRC/MHU (MC_UU__12009), the John Fell Fund (123/737), and the WIMM Strategic Alliance (awards G0902418 and MC_UU_12025). The authors would like to acknowledge M. Oates (Liverpool Bio-Innovation Hub Biobank) for help with CLL sample retrieval. This work was funded by the UK Medical Research Council (MRC core funding of the MRC Human Immunology Unit to J.R.), the Wellcome Trust (grant number 100954 to J.R.), the Swedish Cancer Society (to A.C.), the Swedish Research Council (to A.C.), and a C1 KU Leuven Research Council grant (C14/18/104 to K.D.K.). T.D. was supported by the Wellcome Trust Infection, Immunology & Translational Medicine doctoral programme (grant number 105400/Z/14/Z). A.S. is partly funded by the National Institute for Health Research Oxford Biomedical Research Centre. The views and opinions expressed are those of the authors and do not necessarily reflect those of the National Institute for Health Research, the UK National Health Service, the UK Department of Health, or the Universities of Oxford and Cambridge. The funders had no role in study design, data collection and analysis, decision to publish, or preparation of the manuscript.

AUTHOR CONTRIBUTIONS

Conceptualization: T.D., R.E.R., A.S., and J.R.; Methodology: T.D., R.E.R., C.C., and A.B.; Validation: T.D. and J.R.; Formal analysis: T.D. and J.R.; Investigation: T.D., B.D., R.E.R., H.T.W.B., and S.S.; Resources: J.K., P.H., and A.S.; Data curation: T.D.; Writing – Original Draft: T.D. and J.R.; Writing –

Review & Editing: all authors; Visualization: T.D. and J.R.; Supervision: J.R., A.C., and K.D.K.; Project administration: J.R.; Funding acquisition: T.D. and J.R.

DECLARATION OF INTERESTS

The authors declare no competing interests.

Received: August 13, 2019
Revised: March 12, 2020
Accepted: April 22, 2020
Published: May 12, 2020

REFERENCES

Ahn, J. (2016). Functional organization of human SAMHD1 and mechanisms of HIV-1 restriction. Biol. Chem. *397*, 373–379.

Alonso, R., López-Guerra, M., Upshaw, R., Bantia, S., Smal, C., Bontemps, F., Manz, C., Mehrling, T., Villamor, N., Campo, E., et al. (2009). Forodesine has high antitumor activity in chronic lymphocytic leukemia and activates p53-independent mitochondrial apoptosis by induction of p73 and BIM. Blood *114*, 1563–1575.

Amir, el-A.D., Davis, K.L., Tadmor, M.D., Simonds, E.F., Levine, J.H., Bendall, S.C., Shenfeld, D.K., Krishnaswamy, S., Nolan, G.P., and Pe'er, D. (2013). viSNE enables visualization of high dimensional single-cell data and reveals phenotypic heterogeneity of leukemia. Nat Biotechnol. *31*, 545–552.

Arpaia, E., Benveniste, P., Di Cristofano, A., Gu, Y., Dalal, I., Kelly, S., Hershfield, M., Pandolfi, P.P., Roifman, C.M., and Cohen, A. (2000). Mitochondrial basis for immune deficiency. Evidence from purine nucleoside phosphorylase-deficient mice. J. Exp. Med. *191*, 2197–2208.

Balakrishnan, K., Nimmanapalli, R., Ravandi, F., Keating, M.J., and Gandhi, V. (2006). Forodesine, an inhibitor of purine nucleoside phosphorylase, induces apoptosis in chronic lymphocytic leukemia cells. Blood *108*, 2392–2398.

Balakrishnan, K., Verma, D., O'Brien, S., Kilpatrick, J.M., Chen, Y., Tyler, B.F., Bickel, S., Bantia, S., Keating, M.J., Kantarjian, H., et al. (2010). Phase 2 and pharmacodynamic study of oral forodesine in patients with advanced, fludarabine-treated chronic lymphocytic leukemia. Blood *116*, 886–892.

Balakrishnan, K., Ravandi, F., Bantia, S., Franklin, A., and Gandhi, V. (2013). Preclinical and clinical evaluation of forodesine in pediatric and adult B-cell acute lymphoblastic leukemia. Clin. Lymphoma Myeloma Leuk. *13*, 458–466.

Baldauf, H.M., Pan, X., Erikson, E., Schmidt, S., Daddacha, W., Burggraf, M., Schenkova, K., Ambiel, I., Wabnitz, G., Gramberg, T., et al. (2012). SAMHD1 restricts HIV-1 infection in resting CD4(+) T cells. Nat. Med. *18*, 1682–1687.

Ballana, E., and Esté, J.A. (2015). SAMHD1: at the crossroads of cell proliferation, immune responses, and virus restriction. Trends Microbiol. *23*, 680–692.

Bantia, S., Miller, P.J., Parker, C.D., Ananth, S.L., Horn, L.L., Kilpatrick, J.M., Morris, P.E., Hutchison, T.L., Montgomery, J.A., and Sandhu, J.S. (2001). Purine nucleoside phosphorylase inhibitor BCX-1777 (Immucillin-H)–a novel potent and orally active immunosuppressive agent. Int. Immunopharmacol. *1*, 1199–1210.

Bantia, S., Ananth, S.L., Parker, C.D., Horn, L.L., and Upshaw, R. (2003). Mechanism of inhibition of T-acute lymphoblastic leukemia cells by PNP inhibitor–BCX-1777. Int. Immunopharmacol. *3*, 879–887.

Bantia, S., Parker, C., Upshaw, R., Cunningham, A., Kotian, P., Kilpatrick, J.M., Morris, P., Chand, P., and Babu, Y.S. (2010). Potent orally bioavailable purine nucleoside phosphorylase inhibitor BCX-4208 induces apoptosis in B- and T-lymphocytes–a novel treatment approach for autoimmune diseases, organ transplantation and hematologic malignancies. Int. Immunopharmacol. *10*, 784–790.

Behrendt, R., Schumann, T., Gerbaulet, A., Nguyen, L.A., Schubert, N., Alexopoulou, D., Berka, U., Lienenklaus, S., Peschke, K., Gibbert, K., et al. (2013). Mouse SAMHD1 has antiretroviral activity and suppresses a spontaneous cell-intrinsic antiviral response. Cell Rep. *4*, 689–696.

Bonifati, S., Daly, M.B., St Gelais, C., Kim, S.H., Hollenbaugh, J.A., Shepard, C., Kennedy, E.M., Kim, D.H., Schinazi, R.F., Kim, B., and Wu, L. (2016). SAMHD1 controls cell cycle status, apoptosis and HIV-1 infection in monocytic THP-1 cells. Virology *495*, 92–100.

Bridgeman, A., Maelfait, J., Davenne, T., Partridge, T., Peng, Y., Mayer, A., Dong, T., Kaever, V., Borrow, P., and Rehwinkel, J. (2015). Viruses transfer the antiviral second messenger cGAMP between cells. Science *349*, 1228–1232.

Clifford, R., Louis, T., Robbe, P., Ackroyd, S., Burns, A., Timbs, A.T., Wright Colopy, G., Dreau, H., Sigaux, F., Judde, J.G., et al. (2014). SAMHD1 is mutated recurrently in chronic lymphocytic leukemia and is involved in response to DNA damage. Blood *123*, 1021–1031.

Coquel, F., Silva, M.J., Técher, H., Zadorozhny, K., Sharma, S., Nieminuszczy, J., Mettling, C., Dardillac, E., Barthe, A., Schmitz, A.L., et al. (2018). SAMHD1 acts at stalled replication forks to prevent interferon induction. Nature *557*, 57–61.

Crow, Y.J., and Manel, N. (2015). Aicardi-Goutières syndrome and the type I interferonopathies. Nat. Rev. Immunol. *15*, 429–440.

Daddacha, W., Koyen, A.E., Bastien, A.J., Head, P.E., Dhere, V.R., Nabeta, G.N., Connolly, E.C., Werner, E., Madden, M.Z., Daly, M.B., et al. (2017). SAMHD1 Promotes DNA End Resection to Facilitate DNA Repair by Homologous Recombination. Cell Rep. *20*, 1921–1935.

Dahbo, Y., and Eriksson, S. (1985). On the mechanism of deoxyribonucleoside toxicity in human T-lymphoblastoid cells. Reversal of growth inhibition by addition of cytidine. Eur. J. Biochem. *150*, 429–434.

Dummer, R., Duvic, M., Scarisbrick, J., Olsen, E.A., Rozati, S., Eggmann, N., Goldinger, S.M., Hutchinson, K., Geskin, L., Illidge, T.M., et al. (2014). Final results of a multicenter phase II study of the purine nucleoside phosphorylase (PNP) inhibitor forodesine in patients with advanced cutaneous T-cell lymphomas (CTCL) (Mycosis fungoides and Sézary syndrome). Ann. Oncol. *25*, 1807–1812.

Eriksson, S., Munch-Petersen, B., Johansson, K., and Eklund, H. (2002). Structure and function of cellular deoxyribonucleoside kinases. Cell. Mol. Life Sci. *59*, 1327–1346.

Feoktistova, M., Geserick, P., and Leverkus, M. (2016). Crystal Violet Assay for Determining Viability of Cultured Cells. Cold Spring Harb Protoc. *2016*, pdb.prot087379.

Frank, D.A., Mahajan, S., and Ritz, J. (1997). B lymphocytes from patients with chronic lymphocytic leukemia contain signal transducer and activator of transcription (STAT) 1 and STAT3 constitutively phosphorylated on serine residues. J. Clin. Invest. *100*, 3140–3148.

Franzolin, E., Salata, C., Bianchi, V., and Rampazzo, C. (2015). The Deoxynucleoside Triphosphate Triphosphohydrolase Activity of SAMHD1 Protein Contributes to the Mitochondrial DNA Depletion Associated with Genetic Deficiency of Deoxyguanosine Kinase. J. Biol. Chem. *290*, 25986–25996.

Gabrio, B.W., Huennekens, F.M., and Nurk, E. (1956). Erythrocyte metabolism. I. Purine nucleoside phosphorylase. J. Biol. Chem. *221*, 971–981.

Gandhi, V., and Balakrishnan, K. (2007). Pharmacology and mechanism of action of forodesine, a T-cell targeted agent. Semin. Oncol. *34* (*6, Suppl 5*), S8–S12.

Gandhi, V., Kilpatrick, J.M., Plunkett, W., Ayres, M., Harman, L., Du, M., Bantia, S., Davisson, J., Wierda, W.G., Faderl, S., et al. (2005). A proof-of-principle pharmacokinetic, pharmacodynamic, and clinical study with purine nucleoside phosphorylase inhibitor immucillin-H (BCX-1777, forodesine). Blood *106*, 4253–4260.

Glavas-Obrovac, L., Suver, M., Hikishima, S., Hashimoto, M., Yokomatsu, T., Magnowska, L., and Bzowska, A. (2010). Antiproliferative activity of purine nucleoside phosphorylase multisubstrate analogue inhibitors containing difluoromethylene phosphonic acid against leukaemia and lymphoma cells. Chem. Biol. Drug Des. *75*, 392–399.

Goldstone, D.C., Ennis-Adeniran, V., Hedden, J.J., Groom, H.C., Rice, G.I., Christodoulou, E., Walker, P.A., Kelly, G., Haire, L.F., Yap, M.W., et al.

(2011). HIV-1 restriction factor SAMHD1 is a deoxynucleoside triphosphate triphosphohydrolase. Nature *480*, 379–382.

Gudas, L.J., Ullman, B., Cohen, A., and Martin, D.W., Jr. (1978). Deoxyguanosine toxicity in a mouse T lymphoma: relationship to purine nucleoside phosphorylase-associated immune dysfunction. Cell *14*, 531–538.

Herishanu, Y., Pérez-Galán, P., Liu, D., Biancotto, A., Pittaluga, S., Vire, B., Gibellini, F., Njuguna, N., Lee, E., Stennett, L., et al. (2011). The lymph node microenvironment promotes B-cell receptor signaling, NF-kappaB activation, and tumor proliferation in chronic lymphocytic leukemia. Blood *117*, 563–574.

Herold, N., Rudd, S.G., Ljungblad, L., Sanjiv, K., Myrberg, I.H., Paulin, C.B., Heshmati, Y., Hagenkort, A., Kutzner, J., Page, B.D., et al. (2017a). Targeting SAMHD1 with the Vpx protein to improve cytarabine therapy for hematological malignancies. Nat. Med. *23*, 256–263.

Herold, N., Rudd, S.G., Sanjiv, K., Kutzner, J., Bladh, J., Paulin, C.B.J., Helleday, T., Henter, J.I., and Schaller, T. (2017b). SAMHD1 protects cancer cells from various nucleoside-based antimetabolites. Cell Cycle *16*, 1029–1038.

Hertzog, J., Dias Junior, A.G., Rigby, R.E., Donald, C.L., Mayer, A., Sezgin, E., Song, C., Jin, B., Hublitz, P., Eggeling, C., et al. (2018). Infection with a Brazilian isolate of Zika virus generates RIG-I stimulatory RNA and the viral NS5 protein blocks type I IFN induction and signaling. Eur. J. Immunol. *48*, 1120–1136.

Hikishima, S., Hashimoto, M., Magnowska, L., Bzowska, A., and Yokomatsu, T. (2007). Synthesis and biological evaluation of 9-deazaguanine derivatives connected by a linker to difluoromethylene phosphonic acid as multi-substrate analogue inhibitors of PNP. Bioorg. Med. Chem. Lett. *17*, 4173–4177.

Hikishima, S., Hashimoto, M., Magnowska, L., Bzowska, A., and Yokomatsu, T. (2010). Structural-based design and synthesis of novel 9-deazaguanine derivatives having a phosphate mimic as multi-substrate analogue inhibitors for mammalian PNPs. Bioorg. Med. Chem. *18*, 2275–2284.

Hofer, A., Crona, M., Logan, D.T., and Sjöberg, B.M. (2012). DNA building blocks: keeping control of manufacture. Crit. Rev. Biochem. Mol. Biol. *47*, 50–63.

Hollenbaugh, J.A., Shelton, J., Tao, S., Amiralaei, S., Liu, P., Lu, X., Goetze, R.W., Zhou, L., Nettles, J.H., Schinazi, R.F., and Kim, B. (2017). Substrates and Inhibitors of SAMHD1. PLoS ONE *12*, e0169052.

Howard, D.R., Munir, T., McParland, L., Rawstron, A.C., Milligan, D., Schuh, A., Hockaday, A., Allsup, D.J., Marshall, S., Duncombe, A.S., et al. (2017). Results of the randomized phase IIB ARCTIC trial of low-dose rituximab in previously untreated CLL. Leukemia *31*, 2416–2425.

Hrecka, K., Hao, C., Gierszewska, M., Swanson, S.K., Kesik-Brodacka, M., Srivastava, S., Florens, L., Washburn, M.P., and Skowronski, J. (2011). Vpx relieves inhibition of HIV-1 infection of macrophages mediated by the SAMHD1 protein. Nature *474*, 658–661.

Inoue, K. (2017). Molecular Basis of Nucleobase Transport Systems in Mammals. Biol. Pharm. Bull. *40*, 1130–1138.

Jia, S., Marjavaara, L., Buckland, R., Sharma, S., and Chabes, A. (2015). Determination of deoxyribonucleoside triphosphate concentrations in yeast cells by strong anion-exchange high-performance liquid chromatography coupled with ultraviolet detection. Methods Mol. Biol. *1300*, 113–121.

Johansson, P., Klein-Hitpass, L., Choidas, A., Habenberger, P., Mahboubi, B., Kim, B., Bergmann, A., Scholtysik, R., Brauser, M., Lollies, A., et al. (2018). SAMHD1 is recurrently mutated in T-cell prolymphocytic leukemia. Blood Cancer J. *8*, 11.

Kicska, G.A., Long, L., Hörig, H., Fairchild, C., Tyler, P.C., Furneaux, R.H., Schramm, V.L., and Kaufman, H.L. (2001). Immucillin H, a powerful transition-state analog inhibitor of purine nucleoside phosphorylase, selectively inhibits human T lymphocytes. Proc. Natl. Acad. Sci. USA *98*, 4593–4598.

Kimball, A.K., Oko, L.M., Bullock, B.L., Nemenoff, R.A., van Dyk, L.F., and Clambey, E.T. (2018). A Beginner's Guide to Analyzing and Visualizing Mass Cytometry Data. J. Immunol. *200*, 3–22.

Kong, Z., Jia, S., Chabes, A.L., Appelblad, P., Lundmark, R., Moritz, T., and Chabes, A. (2018). Simultaneous determination of ribonucleoside and deoxyribonucleoside triphosphates in biological samples by hydrophilic interaction liquid chromatography coupled with tandem mass spectrometry. Nucleic Acids Res. *46*, e66.

Kumar, D., Abdulovic, A.L., Viberg, J., Nilsson, A.K., Kunkel, T.A., and Chabes, A. (2011). Mechanisms of mutagenesis in vivo due to imbalanced dNTP pools. Nucleic Acids Res. *39*, 1360–1371.

Lafarge, S.T., Hou, S., Pauls, S.D., Johnston, J.B., Gibson, S.B., and Marshall, A.J. (2015). Differential expression and function of CD27 in chronic lymphocytic leukemia cells expressing ZAP-70. Leuk. Res. *39*, 773–778.

Laguette, N., Sobhian, B., Casartelli, N., Ringeard, M., Chable-Bessia, C., Ségéral, E., Yatim, A., Emiliani, S., Schwartz, O., and Benkirane, M. (2011). SAMHD1 is the dendritic- and myeloid-cell-specific HIV-1 restriction factor counteracted by Vpx. Nature *474*, 654–657.

Lahouassa, H., Daddacha, W., Hofmann, H., Ayinde, D., Logue, E.C., Dragin, L., Bloch, N., Maudet, C., Bertrand, M., Gramberg, T., et al. (2012). SAMHD1 restricts the replication of human immunodeficiency virus type 1 by depleting the intracellular pool of deoxynucleoside triphosphates. Nat. Immunol. *13*, 223–228.

Landau, D.A., Tausch, E., Taylor-Weiner, A.N., Stewart, C., Reiter, J.G., Bahlo, J., Kluth, S., Bozic, I., Lawrence, M., Böttcher, S., et al. (2015). Mutations driving CLL and their evolution in progression and relapse. Nature *526*, 525–530.

Lentz, S.I., Edwards, J.L., Backus, C., McLean, L.L., Haines, K.M., and Feldman, E.L. (2010). Mitochondrial DNA (mtDNA) biogenesis: visualization and duel incorporation of BrdU and EdU into newly synthesized mtDNA in vitro. J. Histochem. Cytochem. *58*, 207–218.

Li, P., Nijhawan, D., Budihardjo, I., Srinivasula, S.M., Ahmad, M., Alnemri, E.S., and Wang, X. (1997). Cytochrome c and dATP-dependent formation of Apaf-1/caspase-9 complex initiates an apoptotic protease cascade. Cell *91*, 479–489.

Li, N., Zhang, W., and Cao, X. (2000). Identification of human homologue of mouse IFN-gamma induced protein from human dendritic cells. Immunol. Lett. *74*, 221–224.

Maelfait, J., Bridgeman, A., Benlahrech, A., Cursi, C., and Rehwinkel, J. (2016). Restriction by SAMHD1 Limits cGAS/STING-Dependent Innate and Adaptive Immune Responses to HIV-1. Cell Rep. *16*, 1492–1501.

Mann, G.J., and Fox, R.M. (1986). Deoxyadenosine triphosphate as a mediator of deoxyguanosine toxicity in cultured T lymphoblasts. J. Clin. Invest. *78*, 1261–1269.

Markert, M.L. (1991). Purine nucleoside phosphorylase deficiency. Immunodefic. Rev. *3*, 45–81.

Maruyama, D., Tsukasaki, K., Uchida, T., Maeda, Y., Shibayama, H., Nagai, H., Kurosawa, M., Suehiro, Y., Hatake, K., Ando, K., et al. (2019). Multicenter phase 1/2 study of forodesine in patients with relapsed peripheral T cell lymphoma. Ann. Hematol. *98*, 131–142.

Moore, E.C., and Hurlbert, R.B. (1966). Regulation of mammalian deoxyribonucleotide biosynthesis by nucleotides as activators and inhibitors. J. Biol. Chem. *241*, 4802–4809.

Munir, T., Howard, D.R., McParland, L., Pocock, C., Rawstron, A.C., Hockaday, A., Varghese, A., Hamblin, M., Bloor, A., Pettitt, A., et al. (2017). Results of the randomized phase IIB ADMIRE trial of FCR with or without mitoxantrone in previously untreated CLL. Leukemia *31*, 2085–2093.

Nègre, D., Mangeot, P.E., Duisit, G., Blanchard, S., Vidalain, P.O., Leissner, P., Winter, A.J., Rabourdin-Combe, C., Mehtali, M., Moullier, P., et al. (2000). Characterization of novel safe lentiviral vectors derived from simian immunodeficiency virus (SIVmac251) that efficiently transduce mature human dendritic cells. Gene Ther. *7*, 1613–1623.

Oellerich, T., Schneider, C., Thomas, D., Knecht, K.M., Buzovetsky, O., Kaderali, L., Schliemann, C., Bohnenberger, H., Angenendt, L., Hartmann, W., et al. (2019). Selective inactivation of hypomethylating agents by SAMHD1 provides a rationale for therapeutic stratification in AML. Nat. Commun. *10*, 3475.

Ogura, M., Tsukasaki, K., Nagai, H., Uchida, T., Oyama, T., Suzuki, T., Taguchi, J., Maruyama, D., Hotta, T., and Tobinai, K. (2012). Phase I study of BCX1777 (forodesine) in patients with relapsed or refractory peripheral T/natural killer-cell malignancies. Cancer Sci. *103*, 1290–1295.

Poli, J., Tsaponina, O., Crabbé, L., Keszthelyi, A., Pantesco, V., Chabes, A., Lengronne, A., and Pasero, P. (2012). dNTP pools determine fork progression and origin usage under replication stress. EMBO J. *31*, 883–894.

Posmantur, R., Wang, K.K., Nath, R., and Gilbertsen, R.B. (1997). A purine nucleoside phosphorylase (PNP) inhibitor induces apoptosis via caspase-3-like protease activity in MOLT-4 T cells. Immunopharmacology *37*, 231–244.

Powell, R.D., Holland, P.J., Hollis, T., and Perrino, F.W. (2011). Aicardi-Goutieres syndrome gene and HIV-1 restriction factor SAMHD1 is a dGTP-regulated deoxynucleotide triphosphohydrolase. J. Biol. Chem. *286*, 43596–43600.

Ran, F.A., Hsu, P.D., Wright, J., Agarwala, V., Scott, D.A., and Zhang, F. (2013). Genome engineering using the CRISPR-Cas9 system. Nat. Protoc. *8*, 2281–2308.

Rehwinkel, J., Maelfait, J., Bridgeman, A., Rigby, R., Hayward, B., Liberatore, R.A., Bieniasz, P.D., Towers, G.J., Moita, L.F., Crow, Y.J., et al. (2013). SAMHD1-dependent retroviral control and escape in mice. EMBO J. *32*, 2454–2462.

Reichard, P. (1988). Interactions between deoxyribonucleotide and DNA synthesis. Annu. Rev. Biochem. *57*, 349–374.

Rentoft, M., Lindell, K., Tran, P., Chabes, A.L., Buckland, R.J., Watt, D.L., Marjavaara, L., Nilsson, A.K., Melin, B., Trygg, J., et al. (2016). Heterozygous colon cancer-associated mutations of SAMHD1 have functional significance. Proc. Natl. Acad. Sci. USA *113*, 4723–4728.

Reubold, T.F., Wohlgemuth, S., and Eschenburg, S. (2009). A new model for the transition of APAF-1 from inactive monomer to caspase-activating apoptosome. J. Biol. Chem. *284*, 32717–32724.

Rice, G.I., Bond, J., Asipu, A., Brunette, R.L., Manfield, I.W., Carr, I.M., Fuller, J.C., Jackson, R.M., Lamb, T., Briggs, T.A., et al. (2009). Mutations involved in Aicardi-Goutières syndrome implicate SAMHD1 as regulator of the innate immune response. Nat. Genet. *41*, 829–832.

Ringshausen, I., Dechow, T., Schneller, F., Weick, K., Oelsner, M., Peschel, C., and Decker, T. (2004). Constitutive activation of the MAPkinase p38 is critical for MMP-9 production and survival of B-CLL cells on bone marrow stromal cells. Leukemia *18*, 1964–1970.

Rudd, S.G., Tsesmetzis, N., Sanjiv, K., Paulin, C.B., Sandhow, L., Kutzner, J., Hed Myrberg, I., Bunten, S.S., Axelsson, H., Zhang, S.M., et al. (2020). Ribonucleotide reductase inhibitors suppress SAMHD1 ara-CTPase activity enhancing cytarabine efficacy. EMBO Mol. Med. *12*, e10419.

Schaller, T., Pollpeter, D., Apolonia, L., Goujon, C., and Malim, M.H. (2014). Nuclear import of SAMHD1 is mediated by a classical karyopherin $\alpha/\beta1$ dependent pathway and confers sensitivity to VpxMAC induced ubiquitination and proteasomal degradation. Retrovirology *11*, 29.

Schmidt, S., Schenkova, K., Adam, T., Erikson, E., Lehmann-Koch, J., Sertel, S., Verhasselt, B., Fackler, O.T., Lasitschka, F., and Keppler, O.T. (2015). SAMHD1's protein expression profile in humans. J. Leukoc. Biol. *98*, 5–14.

Schneider, C., Oellerich, T., Baldauf, H.M., Schwarz, S.M., Thomas, D., Flick, R., Bohnenberger, H., Kaderali, L., Stegmann, L., Cremer, A., et al. (2017). SAMHD1 is a biomarker for cytarabine response and a therapeutic target in acute myeloid leukemia. Nat. Med. *23*, 250–255.

Schuh, A., Becq, J., Humphray, S., Alexa, A., Burns, A., Clifford, R., Feller, S.M., Grocock, R., Henderson, S., Khrebtukova, I., et al. (2012). Monitoring chronic lymphocytic leukemia progression by whole genome sequencing reveals heterogeneous clonal evolution patterns. Blood *120*, 4191–4196.

Shukla, A., Shukla, V., and Joshi, S.S. (2018). Regulation of MAPK signaling and implications in chronic lymphocytic leukemia. Leuk. Lymphoma *59*, 1565–1573.

Swerdlow, S.H. (2008). WHO classification of tumours of haematopoietic and lymphoid tissues (International Agency for Research on Cancer).

Theiss, J.C., Morris, N.R., and Fischer, G.A. (1976). Pyrimidine nucleotide metabolism in L5178Y murine leukemia cells: deoxycytidine protection from deoxyguanosine toxicity. Cancer Biochem. Biophys. *1*, 211–214.

Uhlén, M., Fagerberg, L., Hallström, B.M., Lindskog, C., Oksvold, P., Mardinoglu, A., Sivertsson, Å., Kampf, C., Sjöstedt, E., Asplund, A., et al. (2015). Proteomics. Tissue-based map of the human proteome. Science *347*, 1260419.

Zimmermann, W., Chen, S.M., Bolden, A., and Weissbach, A. (1980). Mitochondrial DNA replication does not involve DNA polymerase alpha. J. Biol. Chem. *255*, 11847–11852.

STAR★METHODS

KEY RESOURCES TABLE

REAGENT or RESOURCE	SOURCE	IDENTIFIER
Antibodies		
Anti-hSAMHD1 (mouse polyclonal)	Abcam	Cat# ab67820; RRID:AB_2301350
Anti-mSAMHD1 (Rabbit)	Rehwinkel et al., 2013	N/A
Anti-mouse AF594 (goat)	Life technologies	Cat# A-11005; RRID:AB_141372
Anti-Caspase 3	Cell Signaling	Cat# 9661T; RRID:AB_2341188
Anti-cytochrome C	BD	Cat# 556433; RRID:AB_396417
Anti-β-actin HRP	Sigma	Cat# A3854; RRID:AB_262011
Anti-Rabbit HRP	GE-Healthcare	Cat# NA934V; RRID:AB_772206
Anti-Mouse HRP	GE-Healthcare	Cat# NA931V; RRID:AB_772210
Anti-gapdh HRP	proteintech	Cat# HRP-60004; RRID:AB_2737588
Anti-COX IV	Cell Signaling	Cat# 11967S; RRID:AB_2797784
Anti-HA HRP	Cell Signaling	Cat# 2999S; RRID:AB_1264166
Anti-PARP	Cell Signaling	Cat# 9542P; RRID:AB_2160739
Anti-BrdU AF488	Biolegend	Cat# 364106; RRID:AB_2564500
Bacterial and Virus Strains		
Biological Samples		
Human peripheral blood mononuclear cells (PBMCs)	ADMIRE and ARCTIC studies (Howard et al., 2017; Munir et al., 2017)	N/A
Human fibroblasts	Yanick Crow Laboratory	N/A
Chemicals, Peptides, and Recombinant Proteins		
Hydroxyurea	Sigma	Cat# H8627-5G
2′-deoxyadenosine monohydrate	Sigma	Cat# D8668-1G
2′-deoxythymidine	Sigma	Cat# T1895-1G
2′ deoxyguanosine	MedChemexpress LLC	Cat# HY-17563
2′-deoxycytidine hydrochloride	MedChemexpress LLC	Cat# HY-17564
Etoposide	Sigma	Cat# E1383-25MG
Forodesine hydrochloride	MedChemexpress LLC	Cat# HY-16209
Benzonase	Sigma	Cat# E8263-5KU
YOYO-3 Iodide	Life technologies	Cat# Y3606
Pepsin	Sigma	Cat# 77160
FxCycle PI/RNase Staining Solution-100 mL	Life technologies	Cat# F10797
Protease inhibitors	Cell signaling	Cat# 871S
Phosphatase inhibitors	Sigma	Cat# P0044
Sample buffer	Life technologies	Cat# NP0007
Novex protein standard	Life technologies	Cat# LC5800
MOPS running buffer	Life technologies	Cat# NP001
MES running buffer	Life technologies	Cat# NP001
NuPAGE Antioxidant	Life technologies	Cat# NP0005
Ponceau	Sigma	Cat# P7170
Tween20	Sigma	Cat# P1379
ECL	Perkin Elmer	Cat# NEL104001EA
ECL prime	Sigma	Cat# GERPN2232
Digitonin	Life technologies	Cat# BN2006
Crystal violet	Sigma	Cat# HT90132

(*Continued on next page*)

Continued

REAGENT or RESOURCE	SOURCE	IDENTIFIER
Propidium iodide	Sigma	Cat# P4170
4-12% Bis Tris protein gel	Life technologies	Cat# NP0321BOX
FcR block	eBiosciences	Cat# 14-9161-73
Aphidicolin	Insight Biotechnology Ltd	Cat# sc-201535
Menadione	Fluorochem	Cat# 049845-1g
MethoCult	Stemcell technologies	Cat# 04100
Glutamax	Thermofisher	Cat# 35050061
IMDM	Thermofisher	Cat# 12440053
Primocin	Invivogen	Cat# ant-pm-1
Anti-Annexin V AF488	Life technologies	Cat# A13201
Critical Commercial Assays		
FITC annexinV / 7AAD detection kit	Biolegend	Cat# 640922
CellTiter-Glo Luminescent Cell Viability Assay	Promega	Cat# G7571
Caspase Glo 3/7	Promega	Cat# G6320
TaqMan Universal PCR Master Mix	Thermofisher	Cat# 4304437
ROS-Glo H_2O_2 assay	Promega	Cat# G8820
Deposited Data		
CyTOF data	This paper	FlowRepository: FR-FCM-Z2JH
Experimental Models: Cell Lines		
Human: HeLa cells	Michael Way Laboratory	N/A
Human: MDA MB231 cells	Alison Banham Laboratory	N/A
Human: B16F10 cells	Vincenzo Cerundolo Laboratory	N/A
Human: Jurkat cells	Simon Davis Laboratory	N/A
Experimental Models: Organisms/Strains		
Mouse: *Samhd1*$^{-/-}$: Samhd1$^{tm1.2Crs}$	Rehwinkel et al., 2013	RRID:MGI:5543265
Oligonucleotides		
mSAMHD1 sgRNA1 fwd: CACCGgaggaactggtagctgtaca	This paper	N/A
mSAMHD1 sgRNA1 rvs: AAACtgtacagctaccagttcctcC	This paper	N/A
mSAMHD1 sgRNA 2 fwd: CACCgggtgaaccccaagctctt	This paper	N/A
mSAMHD1 sgRNA 2 rvs: AAACaagagcttggggttcaccc	This paper	N/A
Primer 1 fwd: tgacagtttgcatctaacctctg	This paper	N/A
Primer 2 rvs: tgacagtttgcatctaacctctg	This paper	N/A
Primer 3 fwd: tgacagtttgcatctaacctctg	This paper	N/A
Recombinant DNA		
pSIV3+	Nègre et al., 2000	N/A
pSIV4+	Nègre et al., 2000	N/A
pCMV-VSVG	Rehwinkel et al., 2013	N/A
pCSHAwtW	Schaller et al., 2014	N/A
pCSHAk11aW	Schaller et al., 2014	N/A
pCSHAh233aW	Herold et al., 2017a	N/A
p8.91 (packaging plasmid)	Rehwinkel et al., 2013	N/A
pMSCVpuro-mSAMHD1	This paper	N/A
pMSCVpuro	Clontech	Cat# 634401

(*Continued on next page*)

Continued

REAGENT or RESOURCE	SOURCE	IDENTIFIER
pX458-Ruby	Hertzog et al., 2018	N/A
pX458	Ran et al., 2013	Addgene Plasmid #48138
pX458-Ruby-sgRNA-1	This paper	N/A
pX458-sgRNA-2	This paper	N/A
Software and Algorithms		
Graph pad prism 7	https://www.graphpad.com	N/A
FlowJo V10	https://www.flowjo.com	N/A
Cytobank	https://www.cytobank.org	N/A
Other		

RESOURCE AVAILABILITY

Lead Contact

Further information and requests for resources and reagents should be directed to and will be fulfilled by the Lead Contact, Jan Rehwinkel (jan.rehwinkel@imm.ox.ac.uk).

Materials Availability

All unique reagents generated in this study are available from the Lead Contact with a completed Materials Transfer Agreement.

Data and Code Availability

The CyTOF data generated during this study are available at FLOWRepository: FR-FCM-Z2JH. The authors declare that all other data supporting the findings of this study are available within the paper and its supplementary information files.

EXPERIMENTAL MODEL AND SUBJECT DETAILS

Mice

Mice were housed and bred under standard conditions at the University of Oxford Biomedical Services Animal Facilities. *Samhd1*$^{-/-}$ mice (C57BL/6N background) were described previously (Rehwinkel et al., 2013). 8-12 week old, male and female C57BL/6N WT and *Samhd1*$^{-/-}$ mice were used to obtain bone marrow for BMDM cultures.

Cells

MEFs were made by standard protocols from either WT or *Samhd1*$^{-/-}$ mice. Bone marrow cells were isolated by standard protocols and, to obtain BMDMs, were grown in Petri dishes for 7 days in R10 medium [Roswell Park Memorial Institute 1640 (RPMI) medium, 10% heat-inactivated fetal calf serum (FCS), 100 U/ml penicillin and 100 μg/ml streptomycin (P/S), 2 mM L-Glutamine] supplemented with 20% L929 conditioned medium and used on day 7. Human fibroblasts from AGS patients were provided by Y. Crow and G.I. Rice. MEFs were cultured in D10 medium [Dulbecco's modified Eagle medium (DMEM) containing 10% heat-inactivated FCS, P/S, 2mM L-Glutamine and 20 mM HEPES buffer]. Human fibroblasts, HeLa, B16F10 and MDA MB231 cells were cultured in D10 without P/S. HeLa cells were a gift from M. Way, MDA MB231 cells were from A. Banham and B16F10 cells were provided by V. Cerundolo. Jurkat cells were a gift from S. Davis and originate from the American Type Tissue Collection and were cultured in R10 without P/S. All cells were cultured under 5% CO_2. Human fibroblasts and MEFs were cultured under low oxygen (1.2% O_2).

Samples from Patients with CLL

PBMCs from 14 patients with CLL recruited into the ADMIRE (n = 10) and ARCTIC (n = 4) studies (Howard et al., 2017; Munir et al., 2017) were retrieved from the Liverpool Bio-Innovation Hub Biobank. Genetic characterization of the tumor cells for these patients was previously published (Clifford et al., 2014) and the patients' gender and age at sample collection are indicated in Figure S4A. PBMCs were thawed in R10 with P/S and 50 U/ml of benzonase, washed twice before being counted and plated. For CellTiter-Glo assay, 50,000 cells were plated in U-bottom 96-well plates. CyTOF experiments were performed using 3,000,000 cells in 12-well non-coated tissue culture plates.

Study Approval

Mouse work was performed in accordance with the UK Animals (Scientific Procedures) Act 1986 and institutional guidelines for animal care. This work was approved by a project license granted by the UK Home Office (PPL No. PC041D0AB) and also was approved by the Institutional Animal Ethics Committee Review Board at the University of Oxford.

Informed consent from all patients was obtained in line with the Declaration of Helsinki. The CLL work was covered by the Ethics approval REC 09/H1306/54. Human fibroblasts from patients with AGS were collected with approval by a UK Multicenter Research Ethics Committee (reference number 04:MRE00/19).

METHOD DETAILS

Plasmids

To generate pMSCVpuro-mSAMHD1, mouse *Samhd1* isoform 1 was PCR amplified. A kozak sequence and N-terminal 3xFLAG-tag were introduced by PCR and the PCR product was cloned into pMSCVpuro using the EcoRI site. To generate SAMHD1-deficient mouse cells, pX458-Ruby-sgRNA-1 and pX458-sgRNA-2 were cloned using pX458-Ruby and pX458, respectively, as described before (Hertzog et al., 2018).

dNTP Measurements

Cells from 4 plates (90 × 15 mm) of BMDMs or 3 plates (150 mm × 20 mm) of MEFs were pooled for each sample. Measurements were done on cells from different mice. Cells were treated with deoxynucleosides for a specific time and washed twice with ice-cold NaCl (9 g/L) on ice. Cells were then scraped in 550 μL of ice-cold trichloroacetic acid (15% w/v), $MgCl_2$ (30mM) solution, collected into an Eppendorf tube, frozen on dry ice and stored at −80°C. Cells were thawed on ice and processed as described in Kong et al. (2018). Briefly, the cell suspension was pulse-vortexed (Intellimixer) at 99 rpm for 10 min at 4°C and centrifuged at 20,000 × g for 1 min at 4°C. The resulting supernatant was then neutralized twice with Freon-Trioctylamine mix (78% v/v - 22%, v/v respectively) by vortexing for 30 s and centrifugation at 20,000 × g for 1 min. 475 μL of the aqueous phase was pH-adjusted with 1 M ammonium carbonate (pH 8.9), loaded on boronate columns (Affi-Gel Boronate Gel; Bio-Rad), and eluted with a 50 mM ammonium carbonate (pH 8.9) and 15 mM $MgCl_2$ mixture to separate dNTPs from NTPs. The eluates containing dNTPs were adjusted to pH 4.5 and loaded onto an Oasis weak anion exchange (WAX) SPE cartridge. Interfering compounds were eluted off the cartridges in two steps with 1 mL ammonium acetate buffer (pH-adjusted to 4.5 with acetic acid) and 1 mL 0.5% ammonia aqueous solution in methanol (v/v), and the analytes were eluted from the cartridge with 2 mL methanol/water/ammonia solution (80/15/5, v/v/v) into a glass tube and then evaporated to dryness using a centrifugal evaporator at a temperature below 37°C. The residue was reconstituted in 1250 μL ammonium bicarbonate buffer, pH-adjusted to 3.4 and used for the HPLC analysis as described in Jia et al. (2015). Briefly, nucleotides were isocratically eluted using 0.36 M ammonium phosphate buffer (pH 3.4, 2.5% v/v acetonitrile) as mobile phase. dNTPs were normalized to total NTP pool of the cells.

Viability Assays

CellTiter-Glo (Promega), a luminescence assay that measures ATP levels, was used according to manufacturer instructions to assess viability. To assess cell viability with crystal violet, cells were stained with 0.5% crystal violet for 20 min, washed 3 times with water and dried overnight before being resuspend in 200 μL methanol and absorbance was measured at 570 nm as described in Feoktistova et al. (2016). For analysis of cell death with the Incucyte live-cell analysis system (Sartorius), Yoyo3 iodide viability die was used at 1/8000 final concentration and images were acquired. The Incucyte was also used to measure confluency and acquire bright-field images over time with a 10x objective.

Apoptosis Assays

The Annexin V/7AAD kit was used to detect apoptotic cells by flow cytometry according to the manufacturer's protocol. Caspase 3/7 Glo (Promega) was used to measure caspase 3/7 cleavage. For live cell imaging, BMDMs were cultured in a glass chamber coated with Poly-L-lysine at 37°C and 5% CO_2. Culture media were supplemented with 2.5mM $CaCl_2$, 20 mM HEPES, propidium iodide (3 μl/well) and Annexin V AF488 (1 μl/well). Images were acquired with a Delta Vision microscope with 10x objective lens every 10 minutes for 24 hours.

Cell Cycle Analysis

BMDMs were seeded at 10^6 cells/well in 6-well low attachment plates and were incubated with 10 μM BrdU for 30 min. In pulse chase experiments, cells were incubated with 10 μM BrdU for 15 min, the media was then replaced, and cells were exposed to dG. At appropriate time points, cells were washed and fixed in 70% ethanol and frozen at −20°C. Cells were washed and resuspend in pepsin solution (1 mg/ml in 30 mM HCl) for 30 min at 37°C, spun down and resuspend in 2M HCl for 15 min at room temperature (RT) and washed with PBS. Cells were then blocked with 0.5% BSA, 0.5% Tween-20 in PBS for 30 min at room temperature, washed and resuspend in FACS buffer with α-BrdU AF488 antibody at 1:100 dilution for 30 min at room temperature. Fix Cycle PI/RNase A staining solution was added to the cells for 30 min at RT. Cells were acquired on a BD LSR II flow cytometer.

Clonogenic Assay

Jurkat cells were treated with dG for 20 hours in IMDM with 10% FCS. 1200 cells were then seeded per well in 6 well plates in methylcellulose, semi-solid medium (40% MethoCult, 39% IMDM, 20% FCS, 1% glutamax and primocin at 100 μg/ml) containing dG. After 13 days incubation, cell colonies were counted manually.

CellPress
OPEN ACCESS

Measurement of ROS Production

H_2O_2 production was measured using the ROS-Glo H_2O_2 assay (Promega) according the manufacturer's instructions.

Western Blots

Cells were lysed in NP-40 buffer (150 mM NaCl, 1% NP-40, 50 mM Tris pH 8.0) with protease and phosphatase inhibitors. After 20 min incubation on ice, lysates were centrifuged at 17,000 g for 10 min at 4°C. Supernatant was collected and diluted with sample buffer before denaturation at 94°C for 5 min. Samples were loaded on pre-cast 4%–12% gradient Bis-Tris protein gels that were run with MOPS or MES buffer at 120 V for 2 hours. Transfer to nitrocellulose membranes was performed in transfer buffer (25mM Tris, 192mM glycine, 20% methanol) at 30 V for 3.5 hours. Membranes were blocked in 5% milk powder in Tris buffered saline with 1% Tween-20 (TBST) for 1 hour at room temperature then washed 5 times for 5 min in TBST. Membranes were incubated with primary antibody in 5% milk TBST overnight at 4°C, then washed 5 times for 5 min in TBST. ECL or ECL prime substrates were used for signal detection. In some experiments, membranes were stripped (0.2 M glycine, 0.1% SDS at pH 2.5) for 15 minutes, washed, blocked, and re-probed with a different antibody.

Retroviral Vectors

VSV-G-pseudotyped retroviral vectors were produced by plasmid transfection of 293T cells (Bridgeman et al., 2015). Retroviral infections were performed in the presence of 8 μg/ml polybrene. Bone marrow cells were transduced three times by spin-infection (2500 rpm, 120 min, 32°C, no brakes) on days 1, 2 and 3 of the 7-day differentiation process with the retroviral vector expressing SAMHD1 or a control vector. Cells were seeded into new plates on day 7 and treated with dG on the next day. THP1 cells were transduced by spin infection (2500 rpm, 120 min, 32°C, no brakes), seeded and treated as indicated in the figure legends. Jurkat cells, MDA-MB231 and HeLa cells were transduced by adding viral vectors to the culture medium. VLP_{vpx} and VLP_{ctrl} were generated using the SIVmac gag-pol expression vectors SIV3+ and SIV4+ (Maelfait et al., 2016). Human SAMHD1 expression constructs pCSHAwtW, pCSHAk11aW and pCSHAh233aW were a kind gift from T. Schaller. These were used to generate lentiviruses for reconstitution of Jurkat cells (Bridgeman et al., 2015). Mouse SAMHD1 expression construct pMSCVpuro-mSAMHD1 was used to generate a retroviral vector to transduce bone marrow cells as described earlier (Rehwinkel et al., 2013).

Stimulation, Staining, and Mass Cytometry Analysis of Patient Samples

PBMCs were collected 24 hours after treatment with forodesine and dG, and were processed according to the Fluidigm Maxpar protocol, using Maxpar reagents. Antibodies are listed in the Table S1. Cells were collected in 15 mL falcon tubes and were washed in PBS, using centrifugation at 300 g for 5 min. Cells were resuspend at 10^7/ml in R0 with Cisplatin (1:10,000) and incubated at 37°C for 5 minutes. Cells were washed with R10 and resuspend in Maxpar PBS. Staining was performed on $3*10^6$ cells/tube. Staining with CD56, CD27, CCR4 and CCR7 was done before fixation. Cells were fixed with Maxpar Fix I Buffer at room temperature (RT) for 10 min then washed with Maxpar Cell Staining Buffer (CSB) and spun at 800 g for 5 min. Cells were barcoded (fluidigm barcoding kit) for 30 min at RT, washed twice in CSB, pooled and counted. All further steps were performed on the pooled sample. Cells were blocked in FcR block diluted in CSB (1:10) for 10 min at RT. Surface staining antibody mix was added to blocking solution and incubated for 30 min at RT. Cells were washed in CSB, resuspend in ice cold methanol added dropwise under the vortex and stored at −80°C overnight. Cells were washed twice with CSB and stained with the intracellular antibody mix for 30 min at RT and stained with intercalator overnight. Next day they were washed with CSB and resuspend in water before acquisition on the Helios mass cytometer (Fluidigm). Samples were normalized, concatenated, and de-barcoded using Helios software. Files were analyzed with Cytobank.

Generation of $Samhd1^{-/-}$ B16F10 Cells

sgRNAs were designed to excise exon 2 of mouse *Samhd1* (Gene ID: 56045). Exon 2 is critical to both isoforms of *Samhd1* (see Figure S2D and Rehwinkel et al., 2013). B16F10 cells were co-transfected with pX458-Ruby-sgRNA-1 and pX458-sgRNA-2 plasmids. GFP-Ruby double positive cells were single cell sorted and clones were expanded. A PCR screening approach was used to identify knock-out cells. PCR-1 was designed to amplify a long fragment (709 bp) from the WT allele and a short fragment (350 bp) from the KO allele using primer 1 fwd and primer 2 rvs (see Figure S2D). PCR-2 had a primer located in exon 2 and amplified a fragment (352 bp) only from the WT allele using primer 2 rvs and primer 3 fwd (Table S1).

QUANTIFICATION AND STATISTICAL ANALYSIS

All experiments were performed three times or more independently under similar conditions, unless specified otherwise in figure legends. Statistical significance was calculated by unpaired t test, one-way ANOVA or two-way ANOVA as described in the figure legends; $p < 0.05$ was considered significant. Graph pad prism 7 software was used to generate graphs and to perform statistical analysis.

Cell Reports, Volume *31*

Supplemental Information

SAMHD1 Limits the Efficacy of Forodesine in Leukemia by Protecting Cells against the Cytotoxicity of dGTP

Tamara Davenne, Jenny Klintman, Sushma Sharma, Rachel E. Rigby, Henry T.W. Blest, Chiara Cursi, Anne Bridgeman, Bernadeta Dadonaite, Kim De Keersmaecker, Peter Hillmen, Andrei Chabes, Anna Schuh, and Jan Rehwinkel

Figure S1

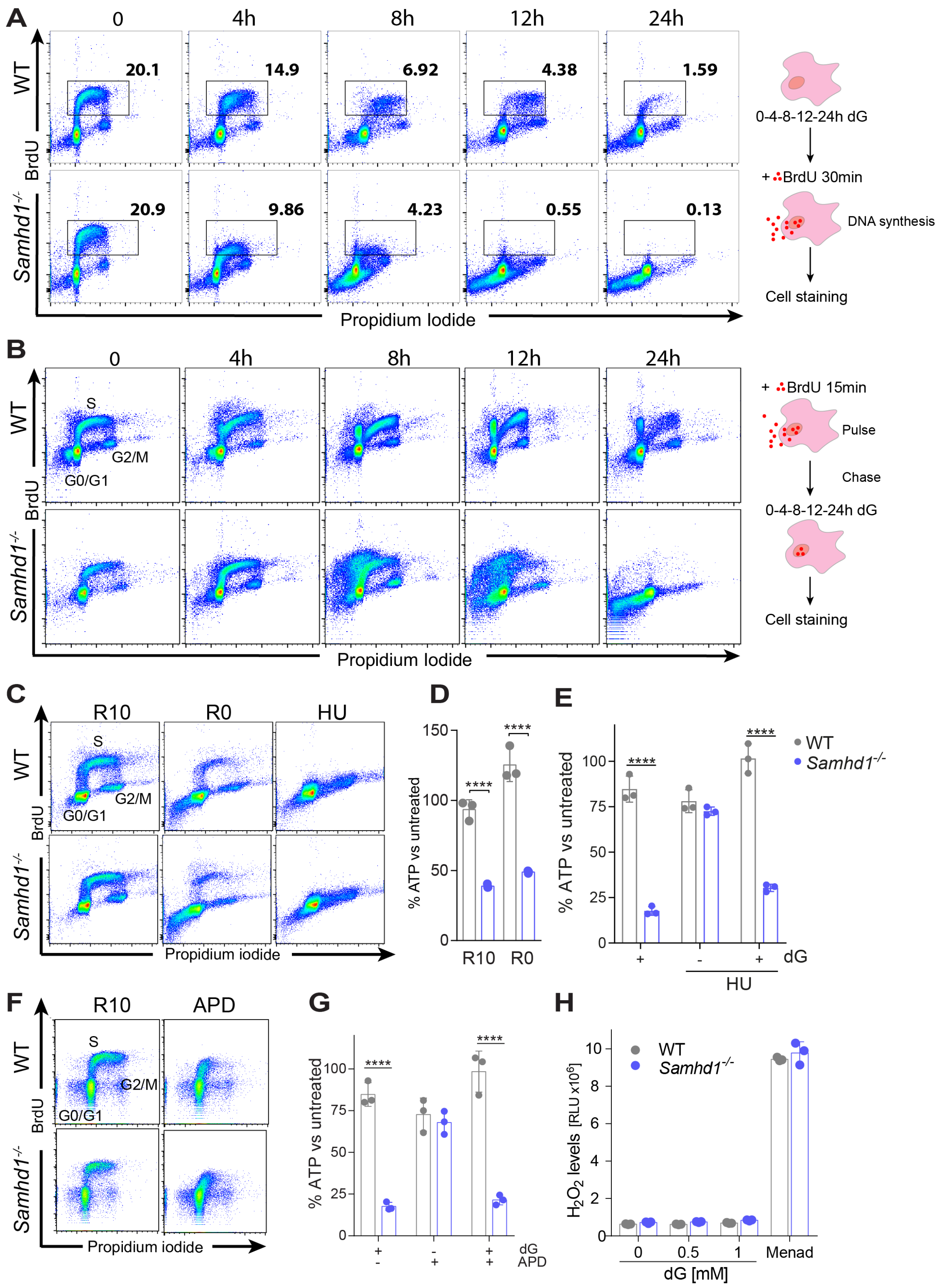

Figure S1. dG-induced cell death in *Samhd1*$^{-/-}$ cells is independent of nuclear DNA replication, related to Figure 2.

(**A**) BMDMs were treated with 0.5 mM dG for the indicated periods of time. Cells were then labelled with BrdU for 30 minutes and fixed. Cells were stained using α-BrdU antibody and PI and analysed by flow cytometry.
(**B**) BMDMs were labelled with BrdU for 15 minutes and then treated with 0.5 mM dG for the indicated periods of time. Cells were then analysed as in (**A**).
(**C-E**) After 7 days of conventional culture, BMDMs were grown in medium containing 10% FCS (R10) or in serum-free medium (R0) for 24 hours. Alternatively, BMDMs were treated with 1 mM hydroxyurea (HU) for 8 hours. (**C**) Cells were analysed as in (**A**). (**D**) Cells were treated with 0.5 mM dG for 24 hours and viability was analysed as described in Figure 1A. (**E**) Cells were treated with 0.5 mM dG for 16 hours and viability was analysed as described in Figure 1A.
(**F-G**) BMDMs were treated or not with 1 μM of aphidicolin (APD) in R10 for 16 hours. (**F**) Cells were analysed as in (**A**). (**G**) Cells were treated with 0.5 mM dG for 24 hours and viability was analysed as described in Figure 1A.
(**H**) H_2O_2 production was measured using the ROS-Glo H_2O_2 assay (Promega). BMDMs were treated with the indicated concentrations of dG for 3 hours. The H_2O_2 substrate solution was then added for 6 hours. Control cells were treated with 20 μM Menadione (Menad) during this period. Luminescence was measured after addition of the detection solution.
Data are representative of three (**A-G**) or two (**H**) independent experiments, respectively. In panels **D-E** and **G-H** data from triplicate measurements are shown with mean ± SD. P-values determined with two-way ANOVA are indicated. **** p<0.0001.

Figure S2

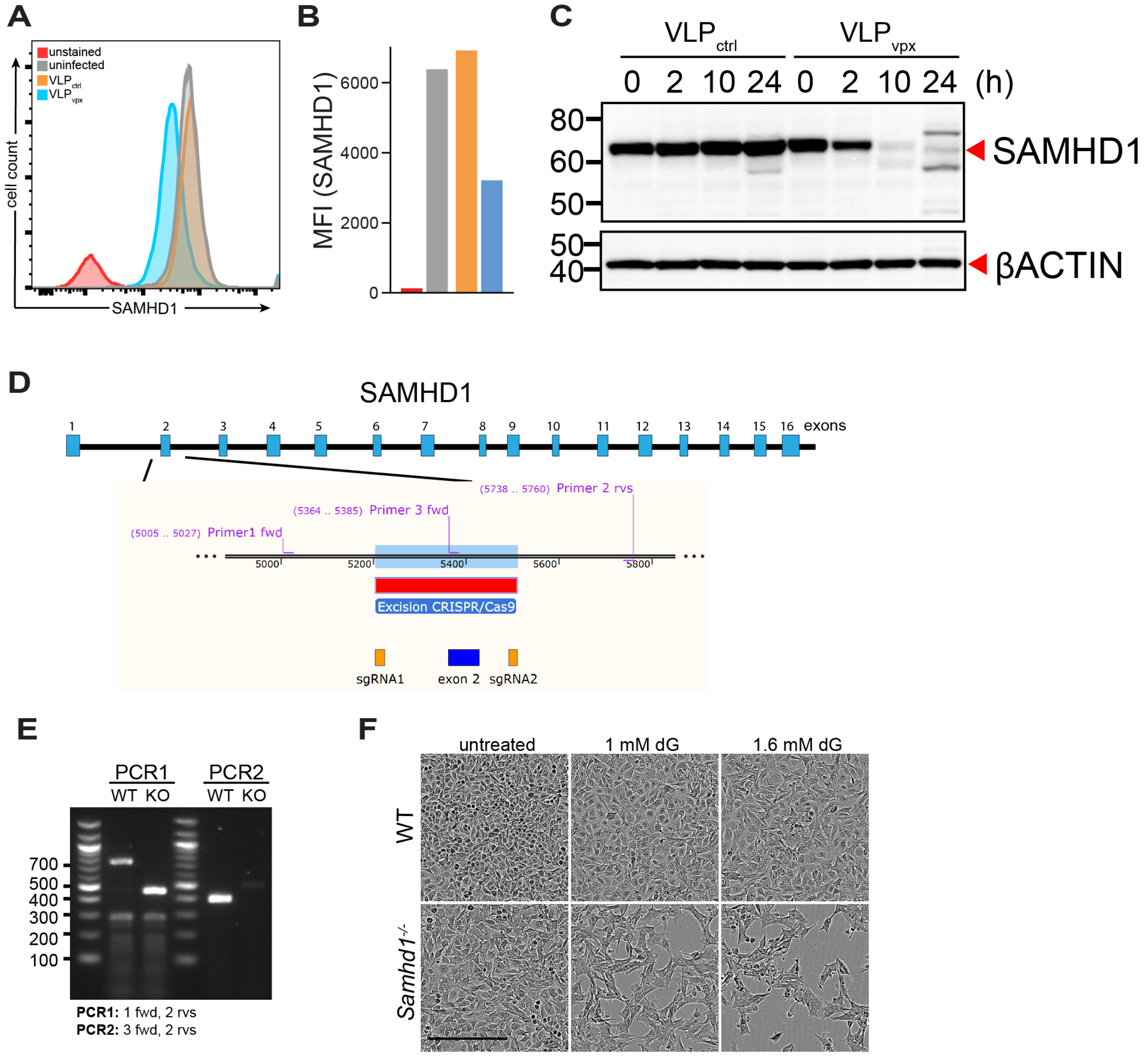

Figure S2. Validation of SAMHD1-deficient cells, related to Figure 3.

(**A,B**) HeLa treated as described in Figure 3A,B were stained with α-SAMHD1 antibody and analysed by flow cytometry. Data are represented as histograms (**A**) and the mean fluorescence intensity (MFI) of the SAMHD1 signal is shown in (**B**).

(C) MDA-MB231 cells were infected with VLPs containing Vpx (VLP_{vpx}) or not (VLP_{ctrl}). SAMHD1 levels in total cell extracts were determined by western blot at the indicated time-points after infection. β-Actin served as a loading control.

(**D,E**) Representation of the CRISPR/Cas9 strategy to generate B16F10 *Samhd1*$^{-/-}$ cells used in Figure 3E-G. The knock-out of *Samhd1* exon 2 was validated by PCR using the indicated primers (**E**).

(**F**) Brightfield images of WT and *Samhd1*$^{-/-}$ B16F10 cells 20 hours after treatment with dG from the experiment in Figure 3G are shown. The scale bar represents 300 μm. Panels **A-C** and **F** are representative of three independent experiments, respectively.

Figure S3

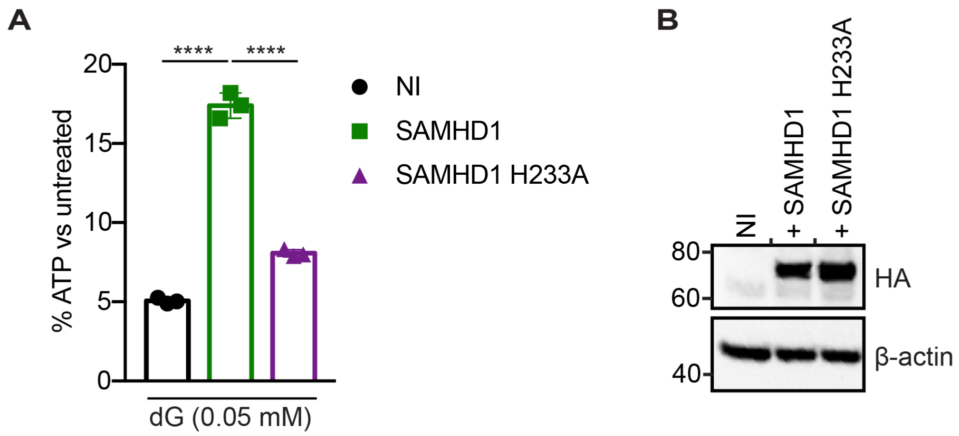

Figure S3. SAMHD1's dNTPase activity is required to protect cells against dG-induced toxicity, related to Figure 3.

(**A**,**B**) Jurkat cells were reconstituted with HA-tagged wild-type or H233A mutant SAMHD1 using a lentivector. Un-infected cells (NI) served as control. (**A**) Cells were then treated with 0.05 mM dG for 48 hours. Cell viability was determined as in Figure 1A. (**B**) SAMHD1 levels in total cell extracts were determined by western blot. β-Actin served as a loading control.

Data are representative of two independent experiments. In panel A, data from triplicate measurements are shown with mean ± SD. P-values determined with two-way ANOVA are indicated. **** $p<0.0001$.

Figure S4

A

Sample ID	Age	Gender	*Del17p*	*TP53*	*SAMHD1*	*del11q (ATM)*	*IgVH status*	Group
ADM224	41	M				y	unmutated	*SAMHD1*^{nonmut}
ADM295	68	M				y	unmutated	
ARC138	66	M				y	unmutated	
ARC554	70	M				y	mutated	
ADM013	72	M		y			missing	*SAMHD1*^{nonmut}
ADM020	69	M		y			unmutated	
ARC334	75	M		y			unmutated	
ADM181	68	M	y	y			unmutated	
ADM216	62	M	y	y			mutated	
ARC603	62	M	y	y			unmutated	
ADM007	59	M			y		unmutated	*SAMHD1*^{mut}
ADM069	59	M			y		unmutated	
ADM210	55	M			y		mutated	
ADM228	57	M			y		unmutated	

B

Sample ID	Exon	Nucleotide	Amino Acid	VAF	SAMHD1 locus
▲ ADM007	exon5	T602A	I201N	0.081	cnLOH
ADM007	exon5	A617G	H206R	0.176	cnLOH
ADM007	exon11	G1165A	A389T	0.492	cnLOH
● ADM069	exon4	G434A	R145Q	0.958	cnLOH
■ ADM210	exon15	1694_1705del	565_569del	0.935	CNLoss
◆ ADM228	exon15	G1715A	W572X	0.982	cnLOH

C

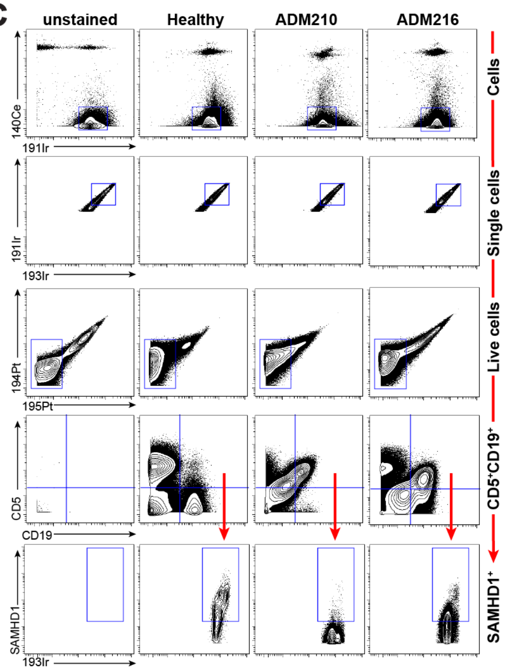

D

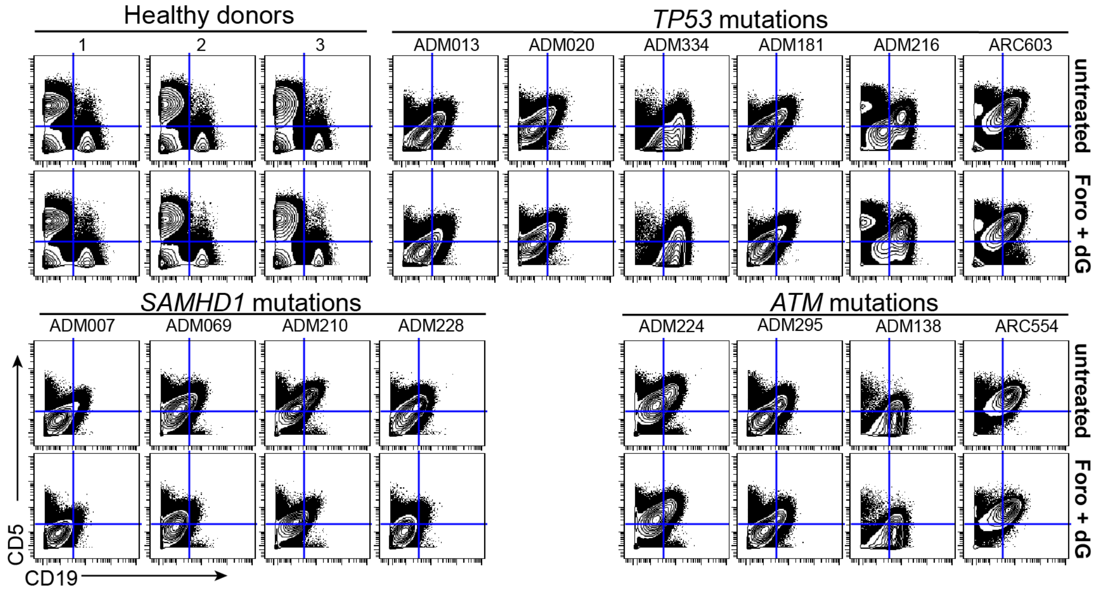

Figure S4. CyTOF analysis, related to Figure 5.

(**A**) List of CLL samples, age of patient at sample collection and mutation status. CLL samples carrying either *TP53* lesions (Del17p or mutation) or *ATM* lesions (Del11q) served as controls for *SAMHD1* mutated samples. M, male; IgVH, immunoglobulin variable heavy chain gene.

(**B**) Details of *SAMHD1* mutations. VAF, Variant allele frequency; cnLOH, copy neutral loss of heterozygosity; CNLoss, copy number loss. The geometrical shapes shown in blue are used in Figure 5 (panels A, C, D, F-H) to identify samples.

(**C**) CyTOF gating strategy for the experiment shown in Figure 5. In brief, the 140Ce channel was used to exclude calibration beads. The 191Ir and 193Ir channels detect cells labelled with an intercalator and were used to identify single cells. The 194Pt and 195Pt channels were used to remove dead (cisplatin-labelled) cells from subsequent analysis of CD5, CD19 and SAMHD1 expression.

(**D**) Expression of CD5 and CD19 is shown as in Figure 5B for all analysed samples.

Figure S5

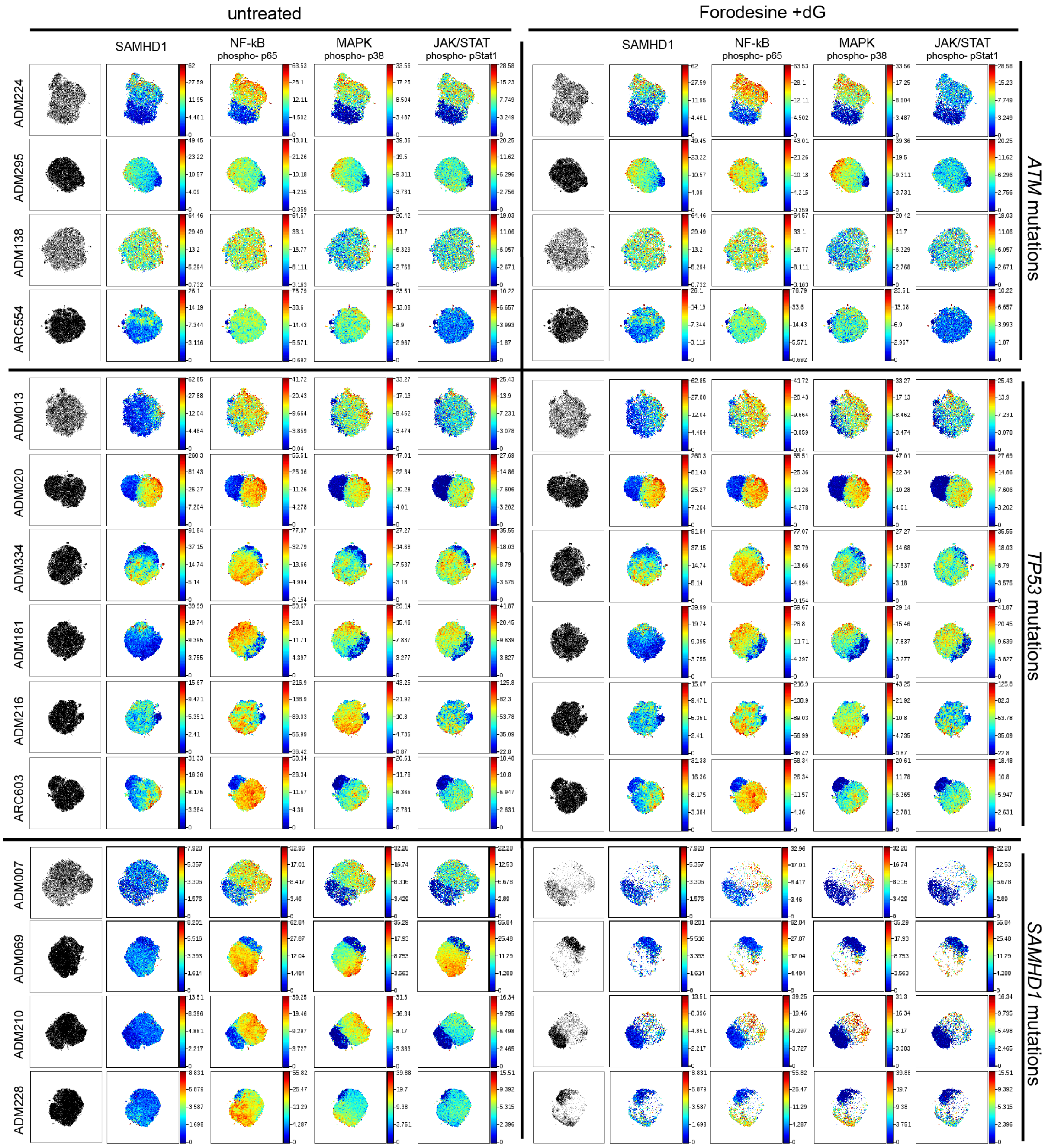

Figure S5. CLL patient cells with *SAMHD1* mutation are more sensitive to Forodesine and dG treatment, related to Figure 5.

CyTOF data from each sample were analysed separately by viSNE analysis as described in Figure 5E. Data from all patients studied are shown; for completeness, we included here those selected for Figure 5E.

Figure S6

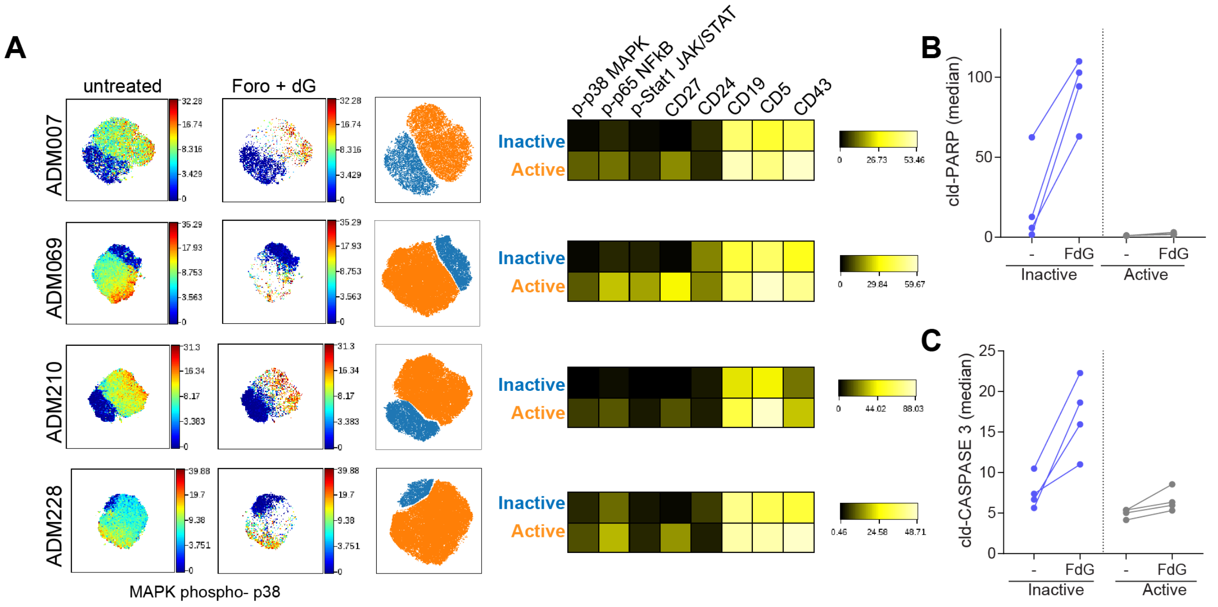

Figure S6. Forodesine and dG induce apoptosis in “inactive” CLL B-cells, related to Figure 5.

(**A**) tSNE plots using live CD5$^+$CD19$^+$ cells from *SAMHD1* mutated patients, coloured by p-p38 levels, were used to manually gate “inactive” [blue] and “active” [orange] cells (left). Expression of selected markers in these sub-populations is shown as a heat map (right).

(**B,C**) The staining for cleaved-PARP (**B**) and cleaved-CASPASE3 (**B**) was analysed in “inactive” and “active” cells. Median values are shown in untreated and treated cells. FdG, treatment with 2 μM forodesine and 20 μM dG.

Table S1. CyTOF antibody panel, related to STAR methods.

Target	Clone	Company	Catalog	Location
CD45	HI30	Fluidigm	3089003B	surface
CD11c	Bu15	Biolegend	337221	surface
CD11b (Mac-1)	ICRF44	Biolegend	301337	surface
CD4	RPA-T4	Fluidigm	3145001B	surface
CD20	2H7	Fluidigm	3147001B	surface
CD56 (NCAM)	NCAM16.2	Fluidigm	3149021B	surface
CD123	6H6	Biolegend	306027	surface
CD27	O323	Biolegend	302839	surface
CD38	HIT2	Biolegend	303535	surface
CD45RA	HI-100	Biolegend	304143	surface
CD14	M5E2	Fluidigm	3160001B	surface
CD23	EBVCS-5	Fluidigm	3164018B	surface
CD197 (CCR7)	G043H7	Fluidigm	3167009A	surface
CD8a	SK1	Fluidigm	3168002B	surface
CD24	ML5	Fluidigm	3169004B	surface
CD3	UCHT1	Fluidigm	3170001B	surface
CD19	HIB19	Biolegend	302247	surface
CD5	UCHT2	Biolegend	300627	surface
HLA-DR	L243	Fluidigm	3174001B	surface
CD194 (CCR4)	205410	Fluidigm	3175021A	surface
CD43	eBio84-3C1	Invitrogen	14-0439-82	surface
CD16	3G8	Fluidigm	3209002B	surface
cld-CASP3	5AE1	Fluidigm	3172023A	intracellular
cld-PARP	F21-852	Fluidigm	3143011C	intracellular
pStat1 [Y701]	4a	Fluidigm	3153005A	intracellular
p38 [T180/Y182]	D3F9	Fluidigm	3156002A	intracellular
SAMHD1	polyclonal	Abcam	Ab67820	intracellular
pNFκBp65 [S529]	K10x	Fluidigm	3166006A	intracellular
